# Inhibition of DYRK1B BY C81 impedes inflammatory processes in leukocytes by reducing STAT3 activity

**DOI:** 10.1007/s00018-025-05579-y

**Published:** 2025-02-22

**Authors:** Sarah Ciurus, Mohammed A. F. Elewa, Megan A. Palmer, Anne Wolf, Mandy Hector, Dominik C. Fuhrmann, Dominique Thomas, Robert Gurke, Martin P. Schwalm, Lena Berger, Thomas J. Zech, Luisa D. Burgers, Rolf Marschalek, Gerd Geisslinger, Stefan Knapp, Thomas Langmann, Franz Bracher, Andreas Weigert, Robert Fürst

**Affiliations:** 1 Institute of Pharmaceutical Biology, Goethe University Frankfurt, Frankfurt, Germany; 2 Institute of Biochemistry I, Goethe University Frankfurt, Frankfurt, Germany; 3 Department of Biochemistry, Faculty of Pharmacy, Kafr El-Sheikh University, Karf El-Sheikh, Egypt; 4 Laboratory for Experimental Immunology of the Eye, Department of Ophthalmology, Faculty of Medicine, University of Cologne, University Hospital Cologne, Cologne, Germany; 5 Centre for Molecular Medicine Cologne (CMMC), University of Cologne, Cologne, Germany; 6 Institute of Clinical Pharmacology, Goethe University Frankfurt, Frankfurt, Germany; 7 Fraunhofer Institute for Translational Medicine and Pharmacology (ITMP), Frankfurt, Germany; 8 Fraunhofer Cluster of Excellence for Immune Mediated Diseases (CIMD), Frankfurt, Germany; 9 Institute of Pharmaceutical Chemistry and Buchmann Institute Molecular Life Sciences, Goethe University Frankfurt, Frankfurt, Germany; 10 Pharmaceutical Chemistry, Department of Pharmacy, Center for Drug Research, Ludwig-Maximilians-University Munich, Munich, Germany; 11 Pharmaceutical Biology, Department of Pharmacy, Center for Drug Research, Ludwig-Maximilians-University Munich, Munich, Germany

**Keywords:** DYRK1B-STAT3 signaling, Kinase inhibitor, Leukocytes, Natural product, Resolution of inflammation

## Abstract

Chronic inflammatory diseases are a significant global burden and are associated with dysregulated resolution of inflammation. Therefore, promoting the process of resolution is a promising therapeutic approach. This study presents the potent anti-inflammatory and pro-resolving effects of a natural product-derived compound called C81. Administration of C81 in a therapeutic window resolved inflammation in the murine imiquimod-induced psoriasis model, and reduced microglial infiltration in a laser-induced choroidal neovascularisation model. Investigations into the underlying mechanisms of C81 identified the DYRK1B/STAT3 axis as a new regulator of inflammatory processes in leukocytes. The inhibition of DYRK1B by C81 resulted in attenuated STAT3 phosphorylation. The depletion of STAT3-regulated gene expression led to the inhibition of leukocyte adhesion and migration due to reduced integrin activation, and in addition to the inhibition of the release of pro-inflammatory mediators such as cytokines and eicosanoids. Importantly, the pro-resolving effects of C81 included the cell type-specific induction of apoptosis in neutrophils and a subsequent increase in efferocytosis. In conclusion, we report the DYRK1B/STAT3 axis as a novel and promising therapeutic target for activating the resolution of inflammation.

**Graphical Abstract:**

**Figure:**
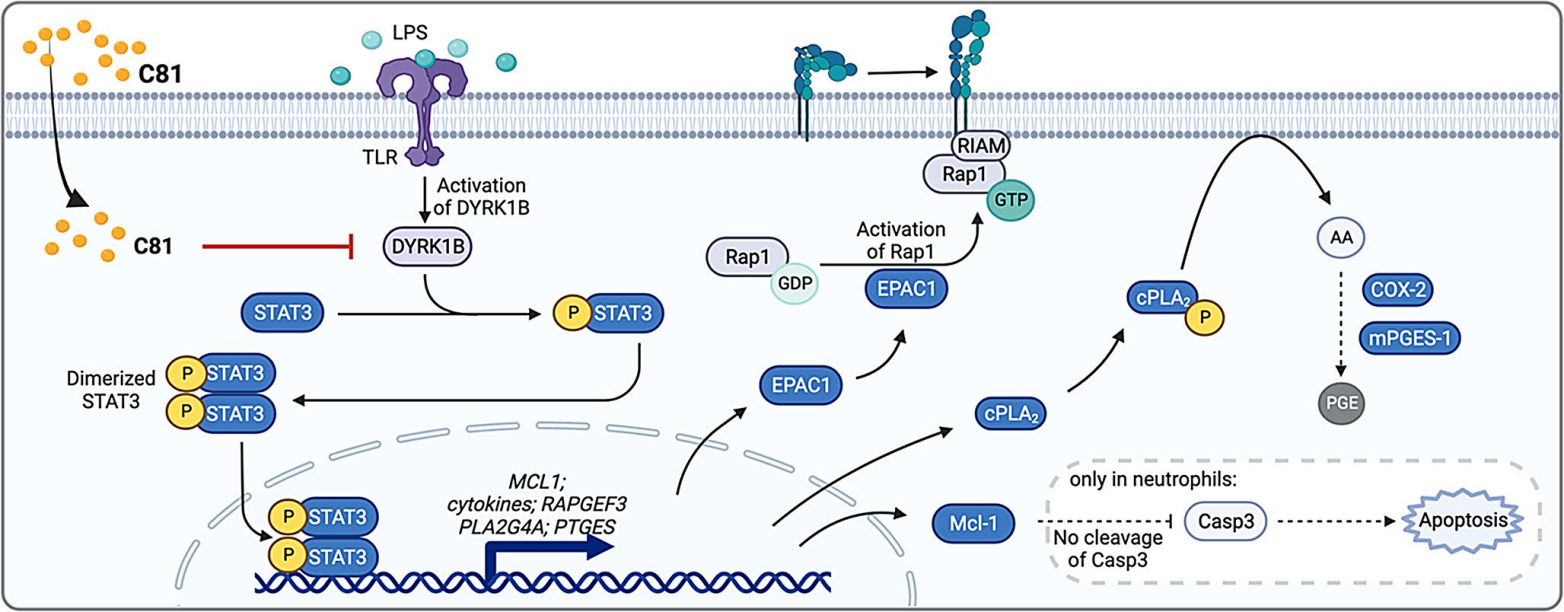
Created with Biorender.com (Science Suite Inc., Toronto, Ontario, Canada)

**Supplementary Information:**

The online version contains supplementary material available at 10.1007/s00018-025-05579-y.

## Introduction

Chronic inflammatory diseases are considered by the World Health Organization (WHO) to be one of the most significant threats to human health [1]. These diseases are associated with dysregulated resolution of inflammation, leading to tissue and/or organ damage due to persistent, low-grade inflammation [2]. The major drawback of the classical therapeutic approaches for the treatment of chronic inflammatory diseases is that they only suppress inflammation and/or the immune system, but do not promote its resolution, resulting only in symptomatic relief [3, 4].

In general, acute inflammation is characterized by a transient upregulation of the inflammatory response to an inflammatory stimulus such as bacterial pathogens. The response involves the release of pro-inflammatory mediators, such as cytokines, chemokines and lipid mediators, and the subsequent attraction of leukocytes towards the inflamed tissue [5–9]. Following integrin-mediated adhesion and transmigration of leukocytes to and through the endothelium, the leukocytes have the primary goal to promote the recruitment of immune cells and eliminate and phagocytose the inflammatory stimulus [10]. Inflammation stops once the threat is removed, and resolution is initiated to prevent the development of acute to chronic inflammation. As part of the resolution process, it is essential that neutrophils, which have largely infiltrated the inflamed tissue, undergo apoptosis and are efferocytosed by macrophages [11, 12]. However, the exact mechanisms of the resolution of inflammation are not yet fully understood and further research is urgently needed for drug development to directly target and activate the resolution of inflammation.

Throughout history, natural products have had a profound impact on drug discovery and development, and natural product research remains a promising resource for uncovering and understanding previously unexplored signaling pathways [13]. C81, a derivative of the natural β-carboline alkaloid annomontine, has recently been shown by our group to exert anti-inflammatory and anti-angiogenic effects on endothelial cells [14–17]. In addition, in earlier studies the compound was characterized as an inhibitor of several kinases, including the dual-specificity tyrosine phosphorylation-regulated kinase 2 (DYRK2) and the CDC2-like kinases 1 and 4 (CLK), which did not appear to be involved in the anti-inflammatory effects of C81 in endothelial cells [14]. In contrast, C81-mediated anti-angiogenic effects, including inhibition of migration, sprouting and proliferation, were indeed mediated by CLK1-4 [17]. CLKs belong to the CMGC superfamily, which also includes the highly similar and conserved kinases of the DYRK family, e.g. DYRK1A, DYRK1B and DYRK2-4, respectively [18, 19]. DYRKs are known to regulate mRNA splicing, cell survival, apoptosis, differentiation and proliferation, and are thought to be involved in various human diseases, including cancer, Alzheimer’s disease, Down syndrome, Parkinson’s disease, osteoarthritis, diabetes and viral infections [20–23]. However, their role in inflammation, and especially in its resolution, is not understood.

The aim of this study was to decipher the pharmacological potential and mode of action of C81, particularly regarding the involvement of its target kinases in inflammatory and resolution mechanisms. In in vivo experiments, specifically in the laser-induced choroidal neovascularization and imiquimod-induced psoriasis model, we investigated the efficacy of the compound. Subsequent in vitro studies included the investigation of cell adhesion, cytokine expression, eicosanoid release, apoptosis and efferocytosis. Analysis of the underlying mechanisms revealed that DYRK1B is the relevant target kinase of C81 for this process in leukocytes. This was verified by pharmacological inhibition, knockdown and overexpression experiments. Furthermore, we report for the first time on the significance of the interplay between DYRK1B and STAT3 in inflammation and its resolution.

## Methods

### Compounds

C81 was kindly provided by the group of Prof. Dr. Franz Bracher (Department of Pharmacy, Center for Drug Research, Ludwig-Maximilians-University Munich, Munich, Germany) and was synthesized and purified as previously described [16]. The chemical structure of C81 can be found in Supplementary Fig. 1. The compound was dissolved in dimethyl sulfoxide (DMSO, Sigma-Aldrich, St. Louis, MO, USA) to 100 mM and stored at -80 °C. The inhibitors AZ-DYRK1B-33 and Stattic were purchased from MedChem Express (Hölzel Diagnostika Handels GmbH, Cologne, Germany), dissolved in DMSO to 10 mM and stored at -80 °C. The stock solutions were freshly diluted for cell culture purposes using cell culture medium without exceeding a final DMSO concentration of 0.1% [v/v]. Lipopolysaccharide (LPS) from *Escherichia coli* O127:B8 was purchased from Sigma-Aldrich (St. Louis, MO, United States). Human recombinant granulocyte-macrophage colony-stimulating factor (GM-CSF), macrophage colony-stimulating factor (M-CSF), IL-4, IFN-g and IL-6 were obtained from PeproTech (Rocky Hill, NJ, USA). Tocilizumab, humanized monoclonal antibody against the interleukin-6 receptor (IL-6R), was obtained from MedChem Express (Hölzel Diagnostika Handels GmbH).

### Animals

For the psoriasis mouse model, 8-week-old male or female C57BL/J mice and for the laser-induced choroidal neovascularization experiment 8- to 10-week-old male or female C57BL/6J mice were used. Mice were obtained from Charles River Laboratories (Wilmington, MA, USA) and were housed on a 12 h light/dark cycle with free access to food and water.

### Psoriasiform dermatitis model

The imiquimod-induced psoriasis mouse model was performed according to previously established protocols [24]. Briefly, the dorsal skin of 8-week-old male or female mice was shaved, and a commercial cream containing 5% imiquimod (Aldara; 3 M Pharmaceuticals, Neuss, Germany) was applied daily to the back skin for six consecutive days at a dose of 62.5 mg. The severity of skin inflammation was assessed using the PASI scoring system, which considers parameters such as skin thickness, redness and scaling over a period of up to 10 days. In addition, mice received subcutaneous injections of either 1 mg/kg of C81 or vehicle (10% DMSO, 90% PBS/Kolliphor EL (90%/10% [v/v]) after 1, 3 and 5 days of imiquimod application. All procedures involving mice were performed in accordance with ethical standards and were approved by the Hessian Animal Care and Use Committee (FU/2064).

### Laser-induced choroidal neovascularization (CNV)

Laser photocoagulation was carried out as described previously [25]. In brief, mice were anesthetized with a mixture of ketamine (100 mg/kg body weight, Ketavet) and xylazine (5 mg/kg body weight, 2% Rompun^Ⓡ^) diluted in 0.9% sodium chloride by intraperitoneal (i.p.) injection and their pupils were dilated with a topical drop of phenylephrine 2.5%/tropicamide 0.5%. A slit-lamp-mounted diode laser system (Quantel Medical Vitra, 532 nm green laser, power 100 mW, duration 100 ms and spot size 100 μm) was used to generate three equal laser burns around the optic nerve in each eye with a cover glass as a contact lens. The animals were randomly assigned to the experimental groups. 1 µl of either 15 µM or 50 µM C81 or corresponding vehicle controls (0.015% or 0.05% DMSO) were injected intravitreally immediately after laser pulse application to reach 3 µM or 10 µM final concentration [26]. After 7 days, mice were euthanized by cervical dislocation and the eyes were enucleated and fixed in 4% of Roti^Ⓡ^-Histofix for 3 h at room temperature (RT). The dissected retinal and RPE/choroidal flat mounts were permeabilized and blocked overnight in Perm/Block buffer (5% normal donkey serum (NDS), 0.2% BSA, 0.3% Triton X-100 in PBS) at 4 °C. The flat mounts were subsequently incubated with a polyclonal rabbit anti-Iba1 antibody (1:500 diluted in Perm/Block, 019-19741, Wako) for 48 h at 4 °C. After washing three times with PBST-X (0.3% Triton X-100 in PBS), the flat mounts were incubated for 1 h with donkey anti-rabbit AlexaFluor™ 594 (1:800 diluted in Perm/Block, A21207, Invitrogen). RPE/choroidal flat mounts were stained in addition with FITC-conjugated isolectin B4 from *Bandeiraea simplicifolia* (1:100 diluted in Perm/Block, L2895, Sigma-Aldrich). After several washing steps, retinal and RPE/choroidal flat mounts were mounted on a microscope slide and embedded with fluorescence mounting medium (Vectashield HardSet H-1400, Vector Labs). Images were taken with a Zeiss Imager.M2 equipped with an ApoTome.2. Morphological analysis of Iba1^+^ mononuclear phagocytes in lasered retinas and RPE/choroidal flat mounts was done using a grid system, and the evaluation of Iba1^+^ area per laser lesion was performed using ImageJ. The average grid crossing points and Iba1^+^ area per laser lesion was calculated. All procedures involving mice were performed in accordance with ethical standards and were approved by the Animal Welfare Commission of North Rhine-Westphalia (NRW) with the permission number Az 81-02.04.2022.A338.

### Isolation of primary human monocytes from peripheral blood mononuclear cells (PBMCs) and isolation of human polymorphonuclear leukocytes (PMNLs)

Primary human leukocytes were freshly isolated from buffy coats from human peripheral blood purchased from Deutsches Rotes Kreuz (Blutspendedienst Baden-Württemberg/Hessen, Institut für Transfusionsmedizin und Immunhämatologie, Frankfurt, Germany). For PBMC and PMNL isolation, leukocytes were isolated by dextran (from *Leuconostoc spp*., Sigma-Aldrich) sedimentation for 30 min followed by a separation by low density gradient centrifugation using lymphocyte separation medium (PromoCell, Heidelberg, Germany). Sedimented PMNLs were collected, and contaminating erythrocytes were lysed by hypotonic lysis in water. Subsequently, PMNLs were washed in ice-cold PBS and finally resuspended in RPMI 1640 (PAN-Biotech, Aidenbach, Germany) containing 10% FCS (Biochrom, Berlin, Germany), 100 U/ml penicillin and 100 µg/ml streptomycin (PAN-Biotech). Cultivation of cells was obtained at 37 °C in an atmosphere of 5% CO_2_ and under constant humidity. For isolation of monocytes, PBMCs sedimented on top of the separation medium were collected, washed twice with ice-cold PBS and the cell pellet was resuspended in starvation medium (RPMI 1640 supplemented with 1% penicillin/streptomycin). The cell suspension was seeded on plates and incubated for at least 1 h at 37 °C. To remove non-adherent lymphocytes, monocytes were washed twice with RPMI 1640 and finally overlaid with full growth medium for monocytes (RPMI 1640 supplemented with 2.5% human serum (HS; purchased from DRK-Blutspendedienst, Frankfurt, Germany) and 1% penicillin/streptomycin).

### Macrophage differentiation

Primary monocytes were differentiated into M1 and M2 macrophages for seven days. For this purpose, cells were treated with 10 ng/ml of GM-CSF (M1) or of M-CSF (M2) for 48 h in full growth medium and were further incubated with the respective colony-stimulating factor for another 72 h after a medium change. Finally, the medium was replenished followed by an incubation for 48 h with GM- or M-CSF in combination with 10 ng/ml of IFN-γ for differentiation into M1 macrophages or 10 ng/ml of IL-4 for differentiation into M2 macrophages.

### Human umbilical vein cell (HUVEC) isolation

HUVECs were isolated from human umbilical veins as previously described (Research Ethics Committee/Institutional Review Board approved waiver W1/21Fü for the use of anonymized human material on 15 September 2021) [27]. Cells were used for experimental purposes exclusively in passage 3 and were cultivated on collagen G (10 µg/ml; Biochrom, Berlin, Germany)-coated plasticware in endothelial growth medium (EASY ECGM, PELOBiotech, Planegg/Martinsried, Germany) supplemented with 10% FCS (Biochrom), 100 U/ml penicillin, 100 µg/ml streptomycin (PAN-Biotech), 2.5 µg/ml amphotericin B (PAN-Biotech) and a supplement mixture (PELOBiotech).

### Cultivation of THP-1 cells

THP-1 (ACC-16) cells, a monocytic leukemia cell line, were purchased from the German Collection of Microorganisms and Cell Cultures (DSMZ, Braunschweig, Germany) and cultivated in RPMI 1640 supplemented with 10% FCS (Biochrom), 100 U/ml penicillin, 100 µg/ml streptomycin (PAN-Biotech). Cells were used up to passage 30 for experimental purposes.

### Cytotoxicity assays

Cell viability assays were performed to exclude any cytotoxicity of C81 on THP-1 cells and primary leukocytes, including monocytes and monocyte-derived M1 and M2 macrophages. For this purpose, every assay was performed in a 96-well plate format (Sarstedt AG & Co., Nümbrecht, Germany) by treating either 50,000 cells per well in case of suspension cells or a confluent layer of adherent cells.

To determine the membrane integrity after C81 treatment, the release of lactate dehydrogenase (LDH) was measured according to the manufacturer’s instructions (CytoTox 96 non-radioactive cytotoxicity assay, Promega, Mannheim, Germany). In brief, cells were treated with the indicated concentrations of C81 or DMSO (0.01% [v/v]) as vehicle control for 24 h. Cell lysis was induced 45 min prior the end of stimulation as a positive control. Afterwards, cell culture supernatants were incubated with substrate solution for 30 min at room temperature and the enzymatic reaction was stopped by addition of stopping solution. Absorbance was measured at 490 nm using a Varioscan Flash microplate reader (Thermo Fisher Scientific, Schwerte, Germany).

A CellTiter-Blue cell viability assay (Promega) was performed to analyze the metabolic activity of compound-treated leukocytes. For this, cells were treated as indicated for 24 h, and 4 h before the end of the incubation time CellTiter-Blue reagent was added to the cells in a ratio of 1:10. Finally, the fluorescence intensity, which arises from the conversion of resazurin to resorufin by viable cells, was determined at 535 nm (ex) and 590 nm (em) using a microplate reader (SPECTRAFluor Plus; Tecan, Männedorf, Switzerland).

Potential C81-induced cell death was examined by using crystal violet staining. In brief, cells were treated with the indicated concentrations of the compound or vehicle (DMSO 0.01%) for 24 h. After the treatment, dead (non-adherent) cells were washed away with PBS+, and remaining viable (adherent) cells were fixed with a methanol-ethanol (2:1) solution, washed with PBS and stained with crystal violet dissolved in 20% methanol. Excessive crystal violet was removed by washing with water, and the cells were left to dry. Subsequently, bound crystal violet was dissolved in 20% acetic acid, and absorbance was measured using a microplate reader (SPECTRAFluor Plus, Tecan) at 590 nm.

### Apoptosis assays

Apoptosis was determined by utilizing flow cytometric analysis by propidium iodide (PI) staining after permeabilization and flow cytometry according to Nicoletti et al. [28], or of cells stained with Annexin V and PI. Staurosporine (MilliporeSigma, Burlington, MA, USA) was used as a positive control. For analysis according to Nicoletti et al. [28], cells were seeded on 24-well plates and were treated with compound or vehicle as indicated. Leukocytes were then collected, washed twice in ice-cold PBS and incubated over night at 4 °C in hypotonic fluorochrome solution containing PI (50 µg/ml; MilliporeSigma), sodium citrate (0.1%; Carl Roth GmbH, Karlsruhe, Germany), and Triton X-100 (0.1%; MilliporeSigma) (HFS-PI). Cells containing sub-diploidic DNA content were measured as apoptotic cells by using FACSVerse flow cytometer (BD Biosciences, Heidelberg, Germany). For Annexin V/PI staining, human neutrophils were treated as indicated for 6 h. Afterwards, the cells were collected, washed with cold PBS and then resuspended in 100 µl annexin binding buffer (BioLegend, Revvity, Waltham, Massachusetts, USA) supplemented with 5 µl of annexin V-FITC (BioLegend). After a 15 min incubation at RT, 400 µl annexin binding buffer and 10 µl PI (20 µg/ml) were added and the cells were measured using flow cytometry. For compensation, staurosporin-treated cells were stained with either annexin V or PI, and unstained and untreated cells were used as the unstained control. The data were analyzed in FlowJo 10.10.0 (FlowJo LLC, BD).

### Cell migration

For analyzing the undirected migration of monocyte-derived M1 and M2 macrophages, a scratch assay was performed. Briefly, a confluent layer of cells was scratched with a pipette tip and was overlaid with either starvation medium (0% migration) or full growth medium containing the indicated concentrations of compound or vehicle. The cells were allowed to migrate for 24 h until the inflicted scratch in the DMSO-treated control was closed (100% migration). Relative migration was quantified by using a Leica DM IL LED inverted microscope (Leica Microsystems) and ImageJ (software version 1.49k). Serum-induced directed migration of monocyte-derived M1 and M2 macrophages was determined by performing a Boyden chamber assay. For this, cells were harvested, stained with Green CMFDA (Cayman Chemical, Ann Arbor, MI, USA), and 1.5 × 10^5^ cells were placed on Transwell inserts (Corning, NY, USA; growth area 0.33 cm^2^, polycarbonate) with a pore size of 8 μm. After treating cells as indicated and applying a chemotactic gradient of 10% HS, the cells were allowed to migrate towards the chemoattractant for 6 h. Non-migrated cells in the upper compartment were removed by using a cotton swab before migrated leukocytes were lysed with RIPA buffer and were quantified using a microplate reader (SPECTRAFluor, Tecan) at 485 nm (ex) and 535 nm (em).

### Cell adhesion assay

HUVECs were seeded on collagen G (10 µg/ml; Biochrom)-coated 48-well plates and were grown until 100% confluency was reached. After treatment of primary monocytes or THP-1 cells as indicated, cells were fluorescently labeled with Green CMFDA (Cayman Chemical), and 7.5 × 10^5^ cells were placed on the endothelial cell monolayer. Primary monocytes and THP-1 cells were allowed to adhere on HUVECs for 4 h and 1 h, respectively. By washing with PBS + non-adherent leukocytes were removed, and remaining attached cells were determined by measuring fluorescence intensity at 485 nm (ex) and 535 nm (em) using a microplate reader (SPECTRAFluor, Tecan).

### Transmigration assay

For further investigation of the leukocyte-endothelial cell interaction, a transmigration assay was performed. Therefore, 1 × 10^5^ HUVECs were seeded on a Transwell membrane (Corning; pore size: 8 μm, growth area: 0.33 cm^2^, polycarbonate) and were grown until 100% confluency. Primary monocytes were treated with the indicated concentrations of C81 for 24 h, stained with Green CMFDA (Cayman Chemical), and 2 × 10^5^ cells were placed on HUVECs. After transmigration for 4 h, cells in the upper compartment of the Transwell system were removed by using a cotton swab. Transmigrated monocytes were lysed with RIPA buffer and the fluorescence intensity was measured by SPECTRAFluor (Tecan) at 485 nm (ex) and 535 nm (em).

### Flow cytometry

The effect of C81 on integrins on the cell surface of leukocytes was analyzed by flow cytometry. For this purpose, primary monocytes and THP-1 cells were seeded on 24-well plates and treated as indicated. Cells were harvested, washed with ice-cold PBS and the integrins LFA-1, VLA-4 and Mac-1 were labeled on the cell surface with fluorochrome-coupled antibodies according to the manufacturer’s instruction. The following antibodies were purchased from BD Biosciences (San Jose, CA, United States): FITC mouse anti-human CD11a (LFA-1), PE mouse anti-human CD49d (VLA-4) and PE mouse anti-Human CD11b (Mac-1). The activation of LFA-1 on the cell surface of THP-1 was determined by using APC mouse anti-human CD11a/CD18 (clone m24) antibody obtained from BioLegend (San Diego). Protein expression or activation was measured using flow cytometry (FACSVerse, BD Biosciences).

### Efferocytosis assay

PMNLs were labeled using CellTrace™ Far Red (Thermo Fisher Scientific) according to the manufacturer’s instructions and were subsequently stimulated with the indicated concentrations of C81 or vehicle for 6 h. As positive control, staurosporine was used to induce apoptosis. Monocyte-derived M1 macrophages were stained with Green CMFDA (Cayman Chemical). Afterwards, PMNLs and macrophages were co-incubated in a ratio of 3:1 at 37 °C in the dark for 60 min. Efferocytosis was analyzed by flow cytometry (FACSVerse, BD Biosciences). The Green CMFDA and CellTrace™ Far Red double-positive population was quantified as successful engulfment of PMNLs by macrophages.

### Reverse transcription quantitative PCR (RT-qPCR)

RNA was isolated using the RNeasy Mini Kit (Qiagen, Hilden, Germany) according to the manufacturer’s instructions with additional on-column DNA digestion using the RNase-free DNase set (Qiagen). 1 µg of isolated RNA was transcribed into cDNA using SuperScript II reverse transcriptase (Life Technologies, Darmstadt, Germany). Based on the 2^− DDCt^ method, qPCR analysis was performed by using the Power SYBR Green PCR Master Mix (Life Technologies) and a StepOnePlus System (Applied Biosystems, Foster City, CA, USA). *B2M*,* UBC* and *GAPDH* served as housekeeping genes. The mRNA levels of *RAPGEF3* (forward: 5’-ATC TGT CAA CGT GGT GAC CC-3’; reverse: 5’-TGT CCA CAC GCA GGA AAT GA-3’), *IL1B* (forward: 5’-GCT GAT GGC CCT AAA CAG ATC-3’; reverse: 5’-GGT GGT CGG AGA TTC GTA GC-3’), *IL6* (forward: 5’-GGT ACA ATC CTC GAC GGC ATC T-3’; reverse: 5’-GTG CCT CTT TGC TGC TTT CAC-3’), *CXCL8* (forward: 5’-TGG CAG CCT TCC TGA TTT CT-3’; reverse: 5’-TTA GCA CTC CTT GGC AAA ACT G-3’), *PLA2G4A* (forward: 5’-TGA CTT TGC CAC ACA GGA CT-3’; reverse: 5’-AAT GTG AGC CCA CTG TCC AC-3’), *DYRK1B* (forward: 5’- TGC GTA AGC TCT CTG TGG AC-3’; reverse: 5’- ACT GCG CAC GAT GTA GTC AT-3’), *B2M* (forward: 5’-AGA TGA GTA TGC CTG CCG TG-3’; reverse: 5’-ACC TCC ATG ATG CTG CTT ACA-3’), *UBC* (forward: 5’-TCA CCC GTT CTG TTG GCT TA-3’; reverse: 5’-TCC AGC TGT TTT CCA GCA AAG-3’) and *GAPDH* (forward: 5’-CCA CAT CGC TCA GAC ACC AT-3’; reverse: 5’-TGA AGG GGT CAT TGA TGG CAA-3’) were determined.

### Western blot analysis

Cells were washed once with PBS and lysed with RIPA lysis buffer supplemented with phenylmethylsulfonyl fluoride, sodium fluoride, sodium orthovanadate, Complete Mini (Roche, Mannheim, Germany), β-glycerophosphate, sodium pyrophosphate and H_2_O_2_. The protein concentration of each sample was determined using a Pierce BCA Protein Assay Kit (Thermo Fisher Scientific). After adding a pyronin-based SDS sample buffer, the samples were incubated at 95 °C for 5 min for denaturation. Proteins were separated by discontinuous SDS-PAGE (Bio-Rad, Feldkirchen, Germany) and transferred to a polyvinylidene fluoride membrane (PVDF; Bio-Rad) by semidry electroblotting using a Trans-Blot Turbo Transfer System (Bio-Rad). Unspecific binding sites on the membranes were blocked with 5% non-fat dry milk (Blotto; Carl Roth GmbH) or 5% BSA (MilliporeSigma) supplemented with 0.1% Tween-20 (Sigma-Aldrich). For detection of the protein of interest, membranes were incubated with respective antibodies: mouse anti-human mPGES-1 (1:1000; sc-166308), mouse anti-human Mcl-1 (1:1000; sc-12756), rabbit anti-human p-cPLA_2_ (ser505) (1:1000; sc-34391) and mouse anti-human cPLA_2_ (1:500; sc-454) were purchased from Santa Cruz Biotechnology (Heidelberg, Germany). Rabbit anti-human COX-2 (1:1000; #12282), rabbit anti-human caspase 3 (1:750; #9662), mouse anti-human STAT3 (1:1000; #9139), rabbit anti-human p-STAT3 (tyr705) (1:1000; #9131) and rabbit anti-human DYRK1B (1:500; #2703) were purchased from Cell Signaling/New England Biolabs (Frankfurt, Germany). Rabbit anti-human EPAC1 (1:500; 12572-1-AP) was obtained from Proteintech (Planegg-Martinsried, Germany). HRP-conjugated secondary anti-mouse (1:3000; #7076; Cell Signaling) and anti-rabbit (1:3000; #7074; Cell Signaling) antibodies were used for detection. A mouse anti-human b-actin-antibody conjugated with peroxidase (1:100.000; A3854; Sigma Aldrich) and a mouse anti-human GAPDH antibody (1:1000; sc-69778; Santa Cruz Biotechnology) were used for the loading controls. Protein expression was detected by chemiluminescence measurement and was quantified by densitometric analysis using ImageJ (software version 1.49).

### Rap1 pulldown assay

To analyze active Rap1, a pulldown assay based on the detection of the GTP-bound Rap1-GTPase by interaction with the RalGDS protein-binding domain was performed. The Active Rap1 Pull-Down and Detection Kit was purchased from Thermo Fisher Scientific (Schwerte, Germany) and was used according to the manufacturer’s instructions. Therefore, THP-1 cells were treated as indicated and were lysed with 500 µl of Lysis/Binding/Wash buffer supplemented with phenylmethylsulfonyl fluoride, sodium fluoride, sodium orthovanadate and Complete Mini (Roche, Mannheim, Germany). After cell lysis, the protein concentration was quantified using a Pierce BCA Protein Assay Kit (Thermo Fisher Scientific), and 500 µl of lysate (containing 1,000 µg of total protein) was transferred to a spin cup. For the loading control, 40 µg of total protein of each sample was saved. After incubation of the lysate with 20 µg of GST-RalGDS-RBD for 1 h at 4 °C, the resin was washed three times, and 50 µl of 2x reducing sample buffer supplemented with 5% b-mercaptoethanol was added. 25 µl of the eluted samples were applied for SDS-PAGE, and detection was performed as described above.

### NanoBRET assay

The assay was performed as described previously [29]. In brief: Full-length DYRK1A, DYRK1B, DYRK2, CLK1, 2 and 4 were obtained as plasmids cloned in frame with a terminal NanoLuc-fusion (kind gift from Promega). Plasmids were transfected into HEK293T cells using FuGENE HD (Promega) and proteins were allowed to express for 20 h. Serially diluted inhibitor, C81 or AZ-DYRK1B-33, and NanoBRET K10 Tracer (Promega, TracerDB ID: T000008) at the Tracer KD concentration taken from TracerDB (tracerdb.org) were pipetted into white 384-well plates (Greiner Bio-One GmbH, Frickenhausen, Germany) using an Echo acoustic dispenser (Labcyte). The corresponding protein-transfected cells were added and reseeded at a density of 2 × 10^5^ cells/ml after trypsinization and resuspending in Opti-MEM without phenol red (Life Technologies). The system was allowed to equilibrate for 3 h at 37 °C/5% CO_2_ prior to BRET measurements. To measure BRET, NanoBRET NanoGlo Substrate + Extracellular NanoLuc Inhibitor (Promega) was added as per the manufacturer’s protocol, and filtered luminescence was measured on a PHERAstar plate reader (BMG Labtech) equipped with a luminescence filter pair (450 nm BP filter (donor) and 610 nm LP filter (acceptor)). Competitive displacement data were then graphed using GraphPad Prism 9 software using a normalized 3-parameter curve fit with the following equation: Y = 100/(1 + 10^(X-LogIC50)).

### DYRK1B-STAT3 ternary complex assay

The assay was performed as described previously [29]. For ternary complex measurements, DYRK1B and STAT3 were cloned in frame with C and N-terminal NanoLuc or HaloTag, respectively. For the transfections, HEK293T cells were diluted in OptiMEM without phenol red (Life Technologies) to 4 × 10^5^ cells/ml and 38 ml were pipetted into white 384-well plates (Greiner). 1 ml FuGENE HD (Promega) transfection mix containing 30 µl FuGENE HD and 8 ml of each plasmid was prepared per plate, incubated 20 min at RT and 2 µl were pipetted into each well leading to the total assay volume of 40 µl. After incubation at 37 °C/5% CO_2_for 20 h 40 nl HaloTag^®^ NanoBRET™ 618 Ligand (Promega) was added to the cells using an Echo acoustic dispenser (Labcyte) and the cells were incubated additional 20 h at 37 °C/5% CO_2_. 2 h prior to BRET measurement, the compounds were titrated to the cells using an Echo acoustic dispenser and the cells were incubated further at 37 °C/5% CO_2_ to allow complex formation. To measure BRET, NanoBRET NanoGlo Substrate + Extracellular NanoLuc Inhibitor (Promega) was added as per the manufacturer’s protocol, and filtered luminescence was measured on a PHERAstar FSX plate reader (BMG Labtech) equipped with a luminescence filter pair (450 nm BP filter (donor) and 610 nm LP filter (acceptor)). Stimulation data were then graphed using GraphPad Prism 10 software.

### Chromatin immunoprecipitation (ChIP) qPCR

For ChIP analysis of monocyte-derived M1 macrophages, sample preparation was performed as described previously for HUVECs [30]. 2 µg of polyclonal rabbit anti-human STAT3 antibody (10253-2-AP; Proteintech) or the negative control antibody rabbit IgG (C15410206; Diagenode, Belgium) was used. The following primers were designed to bind in closest proximity to the STAT3 binding site in the promoter of the target gene *MCL1* and were used for qPCR analysis: 5’-TGC CGT GCG CAA CCC T-3’ (forward) and 5’-GGA AGT GAG AAG TGG CGA GC-3’ (reverse). Data was normalized to background levels of a sequence of the *GAPDH*-untranslated region by using following primers: 5’-CCA CAT CGC TCA GAC ACC AT-3’ (forward) and 5’-TGA AGG GGT CAT TGA TGG CAA-3’ (reverse).

### Generation of stable transfected THP-1 cells for *DYRK1B* knockdown and overexpression

*DYRK1B* was down- or up-regulated in THP-1 (ACC-16) cells by lentiviral transduction: For down-regulation, IPTG-inducible lentiviral vectors (pLV[shRNA]-LacI: T2A: Puro-U6/2xLacO) were constructed and packaged by VectorBuilder (Chicago, IL, USA) containing the shRNA against human *DYRK1B* (Vector ID: VB230419-1124xhu) and a scramble, non-targeting (NT) shRNA (Vector ID: VB010000-9348prq), respectively. For *DYRK1B* overexpression, the vector N174-MCS (Puro) was a gift from Adam Karpf (Addgene plasmid #81068), and the cDNA sequence of *DYRK1B* (NM_004714) was cloned into the vector. As a control, N174-MCS (Puro) without cDNA insertion was transfected. Virus production was accomplished in HEK293T cells through co-transfection with the appropriate shRNA vector, N174-MCS vector and lentiviral packaging vectors, employing the calcium phosphate method. After 48 h, virus particles were collected for the subsequent transduction of THP-1 cells using the PEG-it VPS Kit (SBI System Biosciences, CA, USA). Stably transfected cell lines were established by subjecting the cells to selection with 1 µg/ml puromycin (InvivoGen, Toulouse, France) for a duration of 2 weeks. Knockdown or overexpression of *DYRK1B* was verified by RT-qPCR.

### Lipid mediator formation (LC-MS/MS analysis)

For determination of lipid mediator formation by macrophages, cell culture supernatants were collected, and cell debris was removed by centrifugation (17,000 x g, 5 min, 4 °C). Supernatants were stored at -80 °C until liquid chromatography-tandem mass spectrometry (LC-MS/MS) analysis. Lipid mediators were analyzed by combining liquid-liquid-extraction (LLE) with LC-MS/MS analysis. In brief, 200 µl sample was spiked with 20 µl methanolic IS working solution and 20 µl MeOH, as well as 100 µl 0.15 M EDTA solution. These mixtures were extracted twice with 600 µl ethyl acetate in amber tubes. The upper phase was combined after vortexing with subsequent centrifugation (3 min at 20,000×g) and evaporated at 45 °C under a gentle stream of nitrogen in amber glass vials. The extract was reconstituted in 50 µl MeOH/water (70:30, v/v) containing 0.0001% BHT, transferred into an insert and injected into the LC-MS system. Calibration standards and quality control samples were prepared by spiking 200 µl surrogate matrix and with 20 µl of the methanolic standard working solution and processed as described for the samples. The LC-MS system consisted of a triple quadrupole mass spectrometer QTRAP 6500+ (Sciex, Darmstadt, Germany) equipped with a Turbo V Ion Spray source operated in negative electrospray ionization mode and an Agilent 1290 Infinity LC-system with binary HPLC pump, column oven and autosampler (Agilent, Waldbronn, Germany). The chromatographic separation was performed using an Acquity UPLC BEH C18 2.1 × 100 mm column and VanGuard Pre-Column 2.1 × 5 mm (both with a particle size of 1.7 μm, from Waters, Eschborn, Germany). Analytes were eluted with gradient elution using water (solvent A) and acetonitrile (solvent B) with 0.0025% formic acid, respectively. Data acquisition was done using Analyst Software 1.7.1 and quantification was performed with MultiQuant Software 3.0.3 (both Sciex, Darmstadt, Germany), employing the internal standard method (isotope dilution mass spectrometry). Calibration curves were calculated by linear regression with 1/x weighting.

### Statistical analysis

Statistical analysis was performed using the software GraphPad Prism 9.0 (GraphPad Software, Boston, MA, USA). Data of two independently performed experiments were analyzed by either unpaired t-test or two-way ANOVA. For three or more independently performed experiments, data were analyzed by one-way ANOVA and Tukey’s post hoc test. Data are expressed as mean ± standard deviation (SD) and considered as statistically significant when *p* ≤ 0.05.

## Results

### C81 inhibits inflammation and promotes its resolution in vivo

**Fig. 1:**
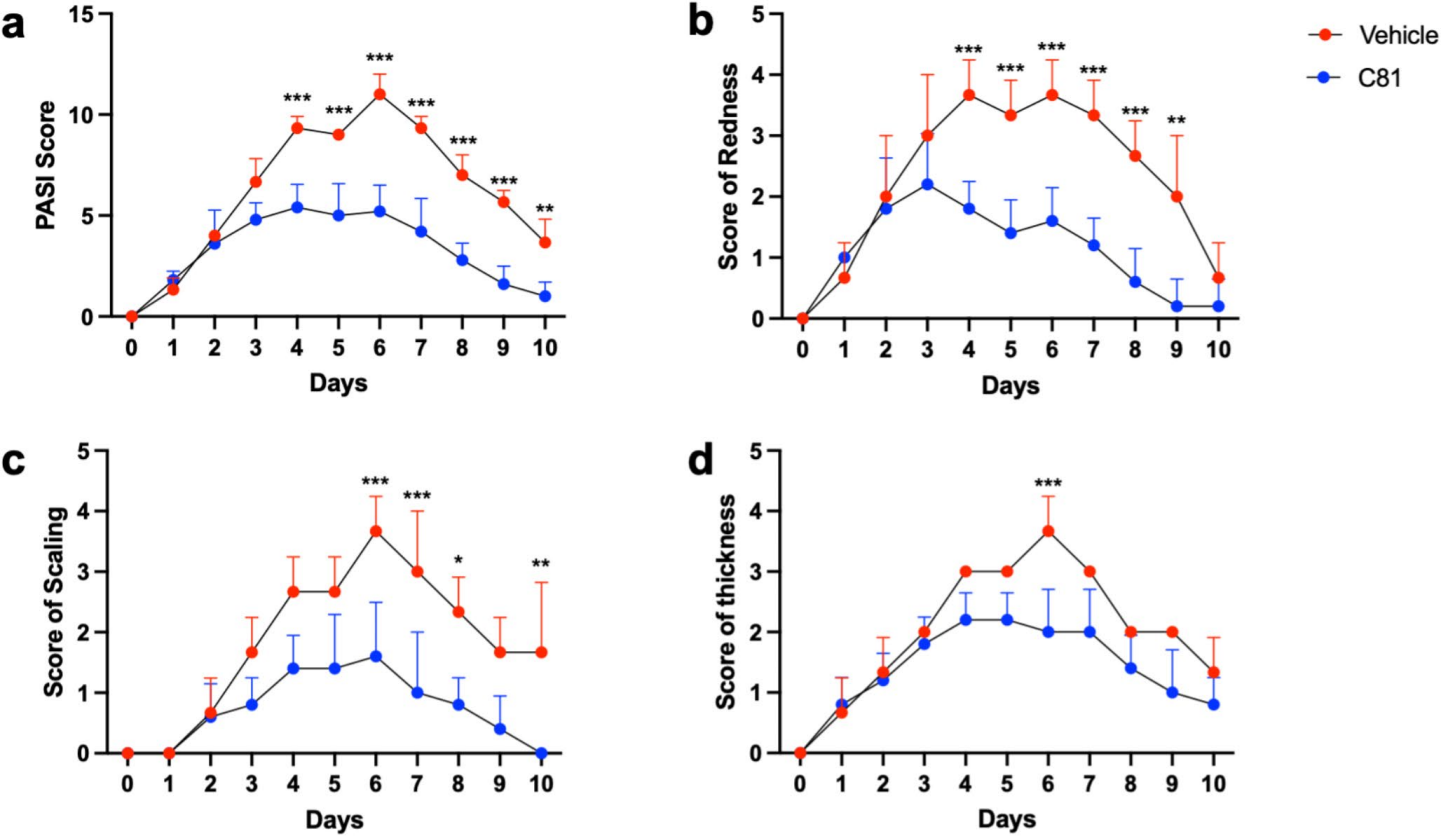
C81 decreases inflammation and promotes resolution of inflammation in the imiquimod-induced psoriasis mouse model. Daily application of imiquimod treatment started on day 0 for 6 consecutive days. C81 (1 mg/kg) or vehicle (10% DMSO, 90% PBS/Kolliphor EL (90%/10% [v/v]) were injected subcutaneously on days 1, 3 and 5. The cumulative Psoriasis Area Severity Index (PASI) score (**a**) was calculated from the individual scores for redness (**b**), scaling (**c**) and thickness (**d**). Scores were analyzed daily for 10 days after first imiquimod application. Data are expressed as mean ± SD. Significance calculated by two-way ANOVA followed by Sidak’s multiple comparison test. * *p* ≤ 0.05, ** *p* ≤ 0.01, *** *p* ≤ 0.001 versus vehicle control for 5 (vehicle) and 5 (C81) individual animals

To investigate the potential anti-inflammatory and pro-resolving effects of C81 in vivo, we utilized an imiquimod-induced psoriasiform inflammation mouse model. Skin inflammation was induced with imiquimod, followed by administration of C81 24 h after the inflammatory reaction was initiated. Based on the response parameters skin redness, scaling and skin thickness, the inflammation reached its peak after 6 days of imiquimod application. The PASI score (Fig. 1a), a cumulative clinical score calculated from these parameters, accordingly, reached its peak with a score of approximately 11 after 6 days. Treatment with C81 (1 mg/kg) strongly reduced redness (approx. 57%) (Fig. 1b), scaling (approx. 57%) (Fig. 1c) and thickness (approx. 46%) (Fig. 1d), which resulted in a dramatic reduction of inflammation by more than 52% in terms of PASI score (Fig. 1a). Importantly, C81 did not only reduce the magnitude of the inflammatory response, but C81-treated mice reached the baseline phenotype of skin redness and thickness around day 9, while inflammation was not resolved in control mice at this point. As a control, the weight of the mice was monitored daily and remained constant throughout the experiment (Supplementary Fig. 2).

**Fig. 2:**
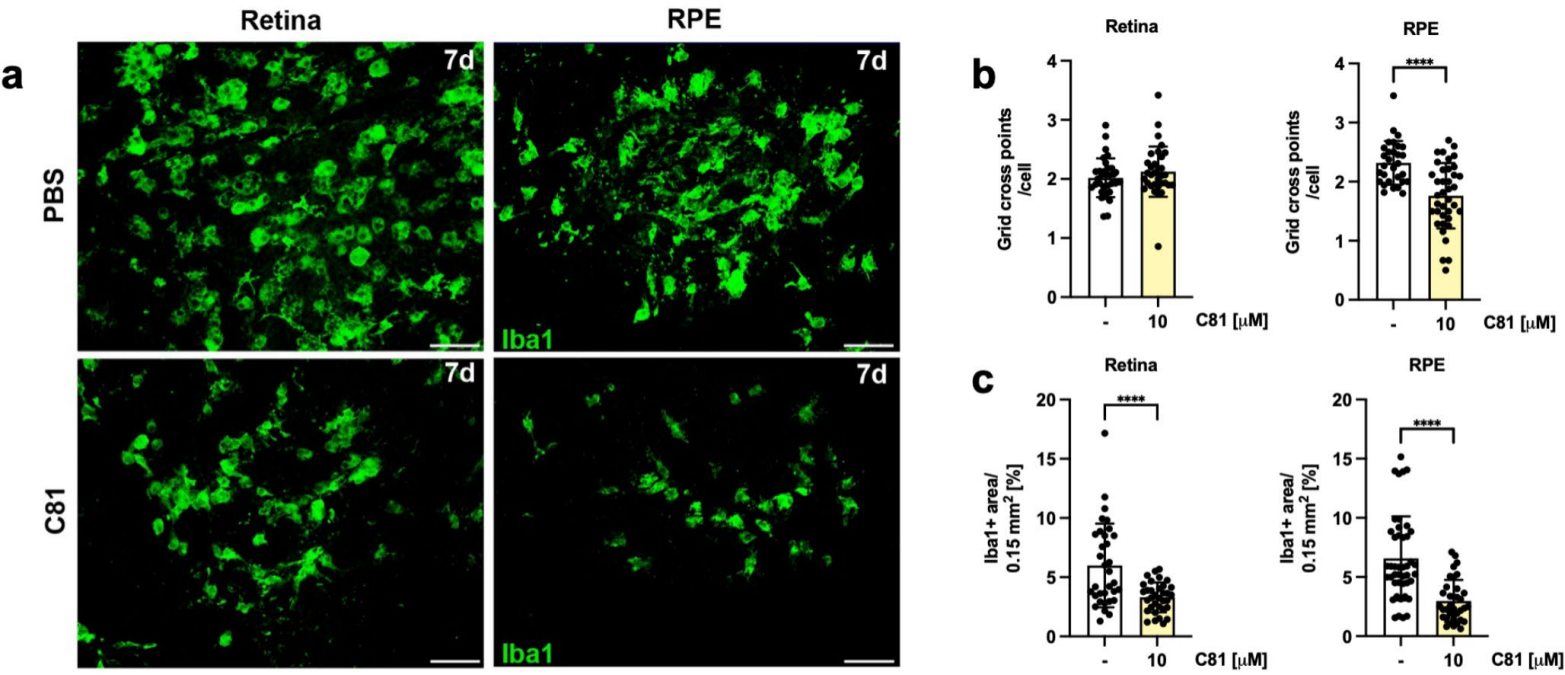
C81 reduces infiltration of Iba1^+^ mononuclear phagocytes in a laser-induced choroidal neovascularization (CNV) model in vivo. C81 (10 µM end concentration in the eye) or vehicle (0.05% DMSO) was injected intravitreally immediately after laser pulse application. After 7 days, morphological changes (**b**) and infiltration (**c**) of Iba1^+^ cells were quantified in retinal and RPE/choroidal flat mounts by immunohistochemistry and fluorescence microscopy (**a**). Scale bar: 50 μm. One representative experiment is shown. *n* = 34–46 retinal spots and *n* = 30–44 RPE/choroidal spots. Data are expressed as mean ± SD. Significance calculated by unpaired t-test. ****p ≤ 0.0001 versus vehicle control for three individual animals

To further study the anti-inflammatory effect of C81 in vivo, we performed a laser-induced choroidal neovascularization experiment (CNV) in mice. Laser burns around the optic nerve caused Bruch’s membrane to rupture and induce inflammation. Infiltration and morphological changes of Iba1^+^ microglia and macrophages in retinas and RPE/choroidal flat mounts were visualized by immunostaining (Fig. 2a). Treatment with C81 did not affect the morphology of Iba1^+^ cells in the retina, but it did in RPE/choroidal flat mounts, inferring reduced microglial activation (Fig. 2b). In addition, the evaluation of Iba1^+^ area per laser lesion, corresponding to microglial infiltration, showed a significant reduction of approx. 45% in retina and approx. 55% in RPE/choroidal flat mounts by treatment with 10 µM of C81 in comparison to the control (Fig. 2c). Even at a lower dose of C81 (3 µM), a reduction of about 30% was observed (Supplementary Fig. 3).

Taken together, these results demonstrate that C81 exerts potent anti-inflammatory and considerably pro-resolving effects in vivo.

### C81 inhibits immune cell functions and interaction with endothelial cells by reduced integrin activation in vitro

To ensure compound-mediated effects in vitro do not derive from cytotoxicity, we first performed cell viability assays. Up to a concentration of 10 µM and a period of 24 h, C81 did not impair metabolic activity, membrane integrity or apoptosis of the human monocytic cell line, THP-1, primary human monocytes and monocyte-derived M1 and M2 macrophages (Supplementary Fig. 4). Additionally, C81 has previously been shown not to cause toxicity in PBMCs up to 10 µM over a period of 72 h [14].

**Fig. 3:**
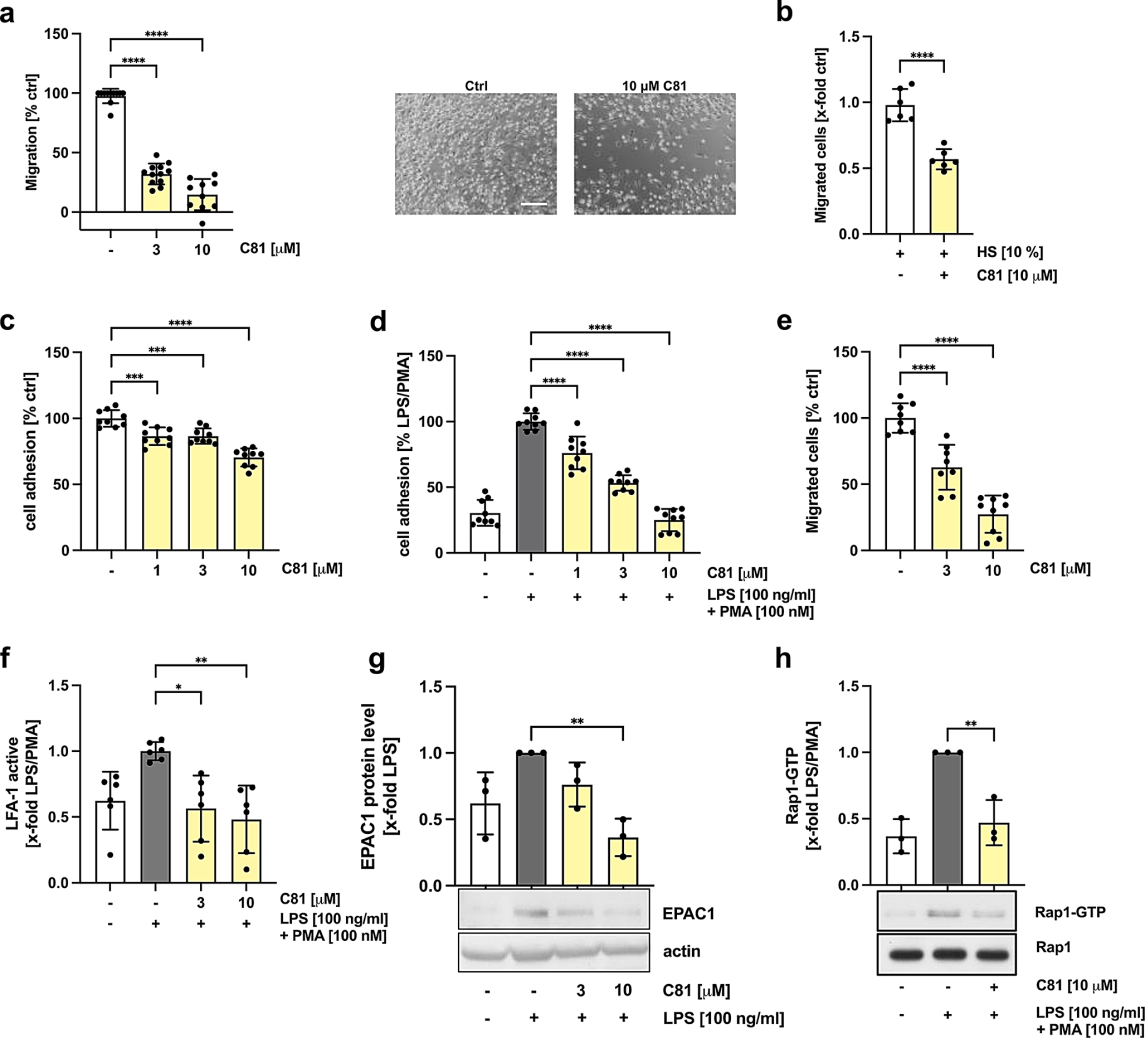
C81 inhibits undirected and serum-induced migration of leukocytes and adhesion to and transmigration through endothelial cells in vitro by attenuating integrin activity. For undirected migration, a confluent layer of monocyte-derived M1 macrophages were treated with C81 or vehicle (DMSO), and a scratch assay was performed. Scale bar: 200 μm (**a**). Serum-induced directed migration of monocyte-derived M1 macrophages was analyzed by a Boyden chamber assay. Fluorescence-labeled cells were allowed to transmigrate for 6 h towards the chemoattractant (10% human serum) in presence of C81 (10 µM) or vehicle (DMSO). Migration was quantified by measuring fluorescence intensity (**b**). For adhesion of primary monocytes (**c**) and THP-1 cells (**d**) to HUVECs, leukocytes were treated with the indicated concentrations of C81 for 24 h. THP-1 cells were additionally treated with 100 ng/ml of LPS for 30 min prior C81 or DMSO treatment, and 100 nM of PMA was added 10 min before the end of the 24 h period. Fluorescence-labeled leukocytes were allowed to adhere for 4 h (primary monocytes) or 1 h (THP-1), and adhesion was analyzed by fluorescence measurement (**c**, **d**). Primary monocytes were treated with C81 for 24 h, fluorescently labeled and placed on an endothelial cell monolayer in a Transwell insert. After 4 h, monocyte transmigration was determined by fluorescence intensity (**e**). THP-1 cells were treated as described previously, and the activation of LFA-1 was quantified by flow cytometry (**f**). EPAC1 protein levels were determined by western blot analysis in THP-1 cells treated with C81 for 24 h and LPS 30 min before compound treatment (**g**). GTP-bound Rap1 was determined by a pulldown assay using GST-RalGDS RBD. Input samples were used as a loading control (**h**). Representative images are shown. Data are expressed as mean ± SD. *n* = 3. Significance calculated by one-way ANOVA followed by Tukey’s post-hoc test. *p ≤ 0.05, **p ≤ 0.01, ***p ≤ 0.001, ****p ≤ 0.0001 versus vehicle control

For in-depth analysis of the anti-inflammatory actions of C81 on leukocytes in vitro, we examined the migration of macrophages and the monocyte-endothelial cell interaction, in particular adhesion and transmigration. To determine the compound’s effect on the undirected and serum-induced directed migration of macrophages, a scratch assay and a Boyden chamber assay were performed, respectively. The undirected migration of M1 and M2 macrophages was already strongly reduced at a concentration of 3 µM, whereas 10 µM of C81 lead to an almost complete inhibition of migration (Fig. 3a, Supplementary Fig. 5). Accordingly, the migration towards a chemotactic stimulus was significantly decreased in the presence of 10 µM of C81 (Fig. 3b). Next, the interaction of primary monocytes or THP-1 cells with HUVECs was investigated, whereby only leukocytes were treated with C81 for 24 h. In addition, THP-1 cells were activated with LPS after pre-treatment with C81 for 30 min, and 10 min prior the end of incubation PMA was added. We found a concentration-dependent and significant suppression of primary monocyte adhesion already significant at 1 µM (Fig. 3c). Furthermore, LPS-PMA induced THP-1 adhesion was also significantly reduced in the presence of 1 µM of C81, while a concentration of 10 µM of C81 fully blocked the interaction of THP-1 cells with HUVECs (Fig. 3d). To assess the transmigration of primary monocytes through an endothelial cell monolayer, a Boyden chamber experiment was carried out. This again showed an inhibition of leukocyte-endothelial cell interaction by C81, as compound-treated primary monocytes displayed a significantly reduced transmigration activity already at 3 µM (Fig. 3e). As leukocyte diapedesis is mediated by integrins [31, 32], we hypothesized that C81 might affect the expression or activation of LFA-1, VLA-4 and Mac-1. Therefore, additional experiments were conducted to analyze the potential effects of C81 on cell surface expression of these markers, but only the VLA-4 expression was slightly altered (Supplementary Fig. 6). Integrins are expressed constitutively on leukocytes and are required to be activated, in particular change their conformation, to promote cell adhesion. LPS and PMA have been shown to induce LFA-1 activity [33, 34] and indeed LPS/PMA treatment resulted in activation of LFA-1, which was strongly reduced in the presence of C81 in THP-1 cells (Fig. 3f). Moreover, LFA-1 activation is mediated by the small GTPase Rap1, which is activated by Rap guanine nucleotide exchange factors (RapGEFs) [35–38]. For monocytes, it was shown that cAMP-dependent EPAC1 is activating Rap1 [34, 39]. Hence, we elucidated the effect of C81 on EPAC1 and found that the compound suppressed *RAPGEF3* (EPAC1) expression at the mRNA(Supplementary Fig. 7) and protein level (Fig. 3g). Consequently, EPAC1-mediated Rap1 activation was reduced upon C81 treatment, which was determined by a Rap1-GTP Pulldown Assay (Fig. 3h). Taken together, C81 attenuates leukocyte diapedesis in vitro by inhibiting integrin activation.

### C81 reduces expression of pro-inflammatory cytokines and eicosanoids in macrophages

**Fig. 4:**
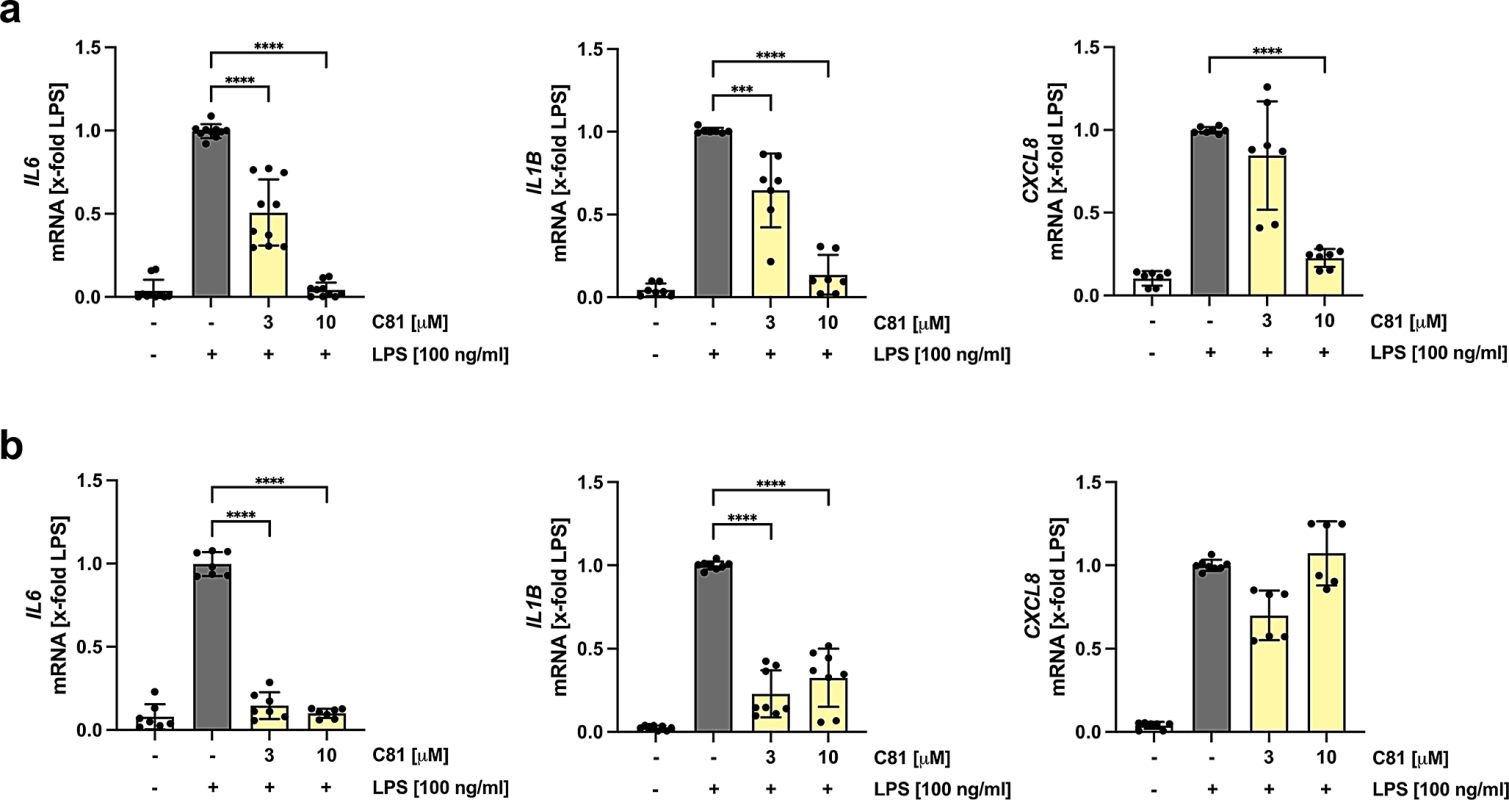
C81 diminishes the expression of pro-inflammatory cytokines. Primary monocyte-derived M1 (**a**) and M2 (**b**) macrophages were treated with LPS for 24 h after a 30 min pre-treatment with C81 or vehicle (DMSO). mRNA expression of *IL6*, *IL1B* and *CXCL8* was determined by RT-qPCR. B2M (**a**) and UBC (**b**) were used as housekeeping genes. Data are expressed as mean ± SD. *n* = 3. Significance calculated by one-way ANOVA followed by Tukey’s post-hoc test. ****p ≤ 0.0001 versus vehicle control

During inflammation, macrophages are the major producers of pro-inflammatory mediators, including cytokines and chemokines. A potential effect of C81 on the expression of these mediators was investigated in monocyte-derived M1 and M2 macrophages on mRNA levels by RT-qPCR. The results showed a significant inhibition of LPS-induced *IL6* and *IL1B* expression already at 3 µM of C81, whereas at a concentration of 10 µM the expression of these cytokines was completely abolished (Fig. 4a, left and middle). Also, the mRNA levels of *CXCL8* (IL-8) were strongly reduced in M1 macrophages (Fig. 4a, right). A similar effect was observed for M2 macrophages, where the expression of *IL6* and *IL1B* was already inhibited at a lower concentration (3 µM) when comparing to M1 macrophages. However, C81 had no impact on *CXCL8* expression (Fig. 4b).

**Fig. 5:**
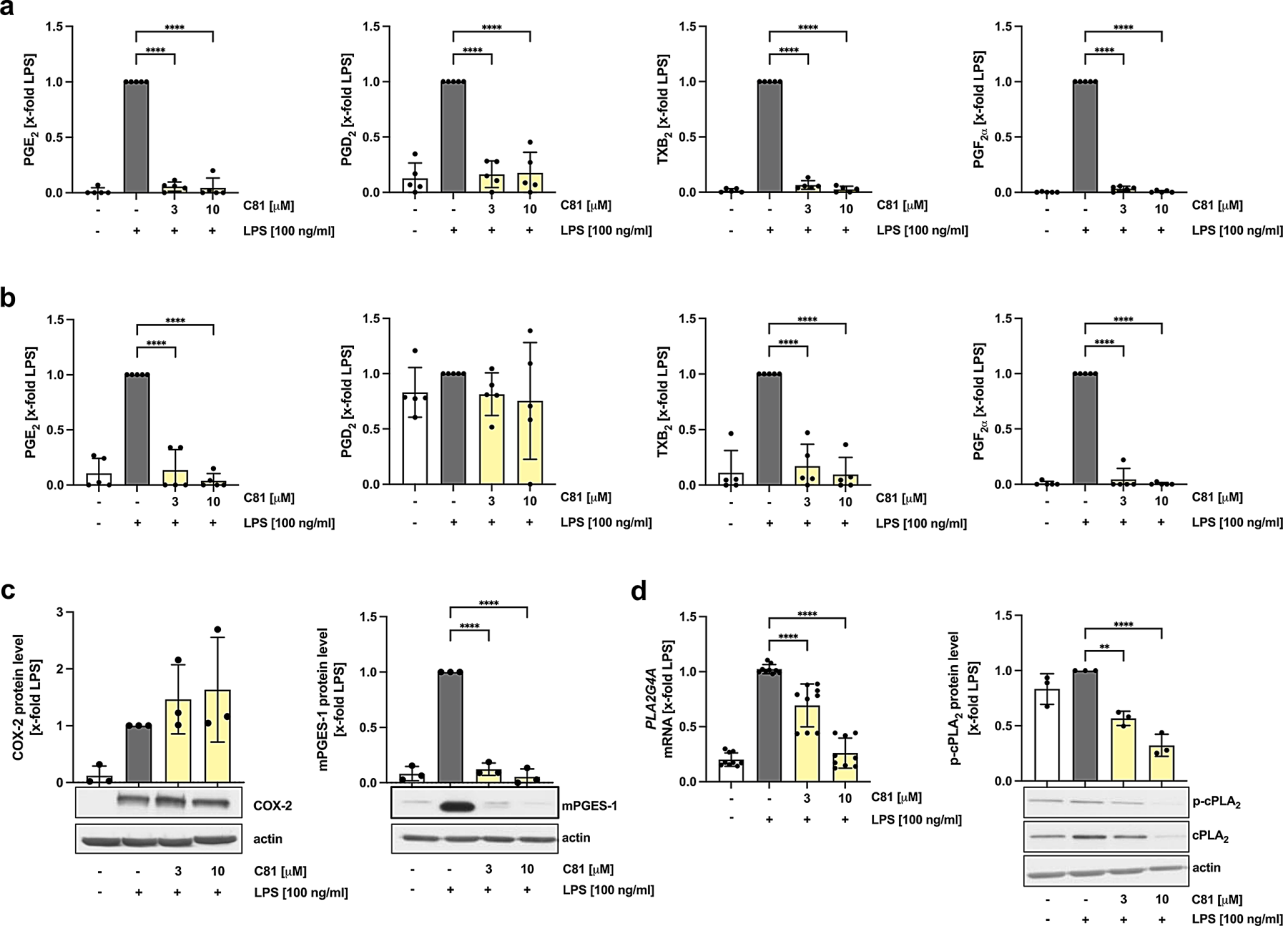
C81 attenuates the release of pro-inflammatory eicosanoids by reducing cPLA_2_ expression. Primary monocyte-derived M1 (**a**, **c**, **d**) and M2 (**b**) macrophages were treated with LPS for 24 h after a 30 min pre-treatment with C81 or DMSO. Release of PGE_2_, PGD_2_, TXB_2_ and PGF_2α_ were quantified in the supernatant by LC-MS/MS. 0 ≙ < LLOQ (20–80 pg/ml) (**a**, **b**). Protein level of COX-2 and mPGES-1 in M1 macrophages was determined by western blot analysis. Actin was used as loading control (**c**). mRNA level of *PLA2GA4* and the protein levels of phospho- as well as total-cPLA_2_ were quantified by RT-qPCR and western blot analysis, respectively. GAPDH was used as housekeeping gene and actin as loading control (**d**). Representative blots are shown. Data are expressed as mean ± SD. (**a**, **b**) *n* = 5, (**c**,** d**) *n* = 3. Significance calculated by one-way ANOVA followed by Tukey’s post-hoc test. ****p ≤ 0.0001 versus vehicle control

Besides cytokines and chemokines, macrophages also release pro-inflammatory lipid mediators, when they are exposed to an inflammatory stimulus. Therefore, the secretion of lipids was examined in the supernatant of M1 and M2 macrophages by LC-MS/MS. Activation of macrophages with LPS led to increased concentrations of PGE_2_, PGD_2_, TXB_2_ and PGF_2a_ expression in M1 macrophages. Surprisingly, incubation with C81, at a concentration of 3 µM already, completely inhibited the production of eicosanoids measured (Fig. 5a). In M2 macrophages the same pattern and intensity of inhibition was observed, except that the release of PGD_2_ was not inducible by LPS and not affected by C81 (Fig. 5b). We hypothesized a compound-derived effect on the expression of cyclooxygenases, which are essential to produce eicosanoids. While we observed a non-significant compound-dependent up-regulating rather than a down-regulating effect on LPS-induced COX2 expression (Fig. 5c, left), C81 led to a dramatic inhibition of mPGES-1 on protein levels (Fig. 5c, right). Of note, mPGES-1 acts downstream of the cyclooxygenases to convert PGH_2_ to PGE_2_. Blocking this enzyme alone would only explain the attenuated PGE_2_ release. To explain an overall inhibition of eicosanoids, upstream mechanisms, such as hydrolysis of membrane phospholipids to arachidonic acid mediated by cPLA_2_, must be altered by C81. Indeed, the LPS-induced expression of cPLA_2_ was inhibited upon C81 treatment on mRNA and protein levels as determined by RT-qPCR and western blotting, respectively (Fig. 5d).

In conclusion, C81 exerts anti-inflammatory properties by attenuating the expression of important pro-inflammatory cytokines and chemokines. Moreover, the compound showed a dramatic inhibitory effect on the secretion of lipid mediators by inhibition of cPLA_2_ expression.

### C81 induces apoptosis only in human neutrophils and promotes efferocytosis

**Fig. 6:**
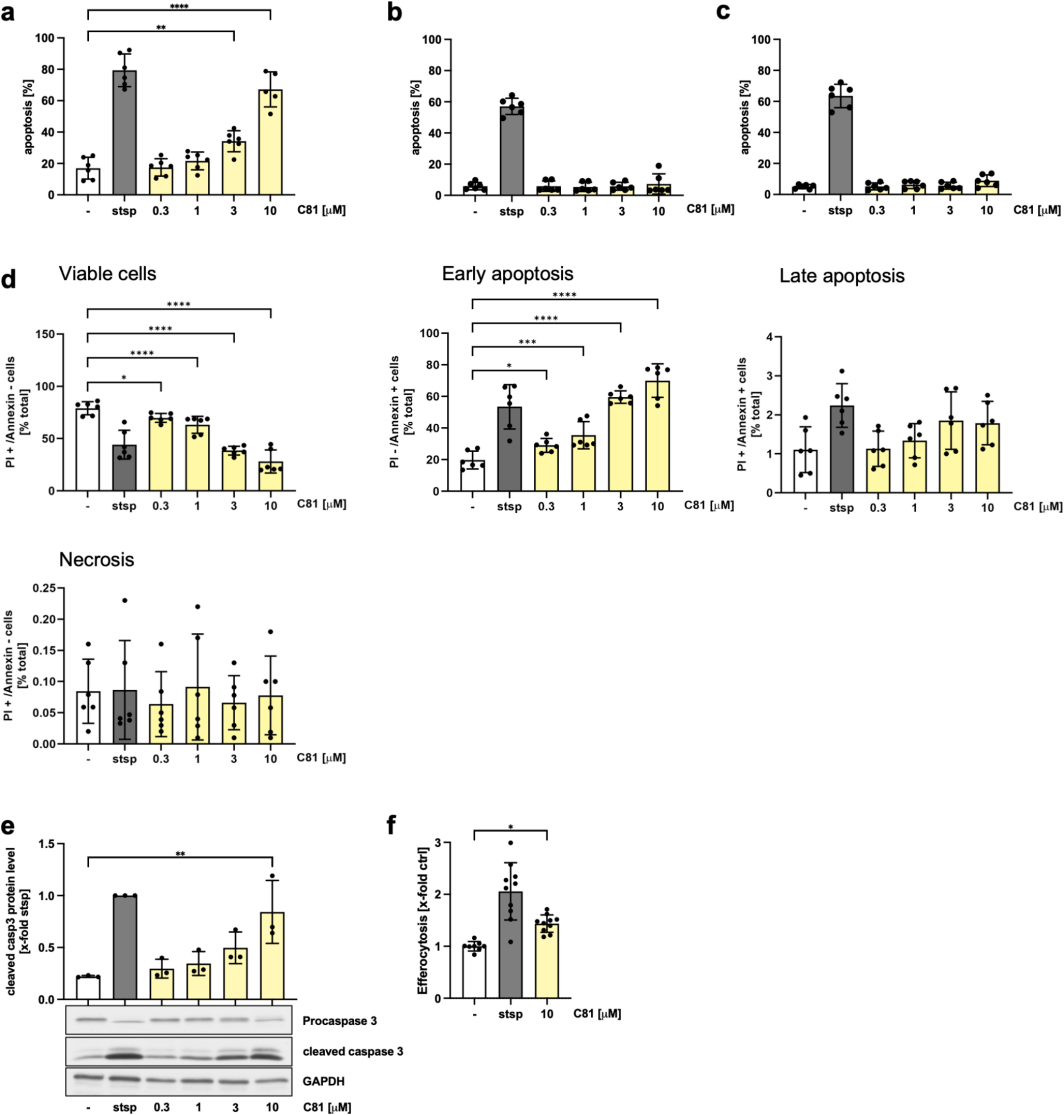
C81 induces apoptosis in human neutrophils and promotes efferocytosis by macrophages. Apoptosis of human neutrophils (**a**), M1 macrophages (**b**) and M2 macrophages (**c**) was determined by propidium iodide staining and flow cytometry (events with sub-diploid DNA content) according to Nicoletti et al., 1991 (**a-c**). Measurement of viable, early and late apoptotic and necrotic neutrophils using annexin V/PI costaining of neutrophils (**d**). Cells were treated for 6 h (neutrophils) or 24 h (macrophages) with the indicated concentrations of C81 or DMSO. Caspase 3 activity in neutrophils was determined by analyzing the cleaved isoform by western blot analysis. GAPDH was used as loading control (**e**). For efferocytosis, human neutrophils were fluorescently labeled with CellTrace™ Far Red and treated with C81 for 6 h. Monocyte-derived M1 macrophages were fluorescently labeled with Green CMFDA. C81-treated neutrophils and non-treated macrophages were co-incubated for 1 h. Engulfment of neutrophils by macrophages was determined by flow cytometry. The double-positive (Green CMFDA + CellTrace™ Far Red) population was quantified as efferocytosis (**f**). Staurosporine (1 µM) was used as a positive control (**a-f**). Data are expressed as mean ± SD. *n* = 3. Significance calculated by one-way ANOVA followed by Tukey’s post-hoc test (**a-c**, **e**, **f**) or two-way ANOVA followed by Dunnett’s post-hoc test (**d**). *p ≤ 0.05, **p ≤ 0.01, ***p ≤ 0.001, ****p ≤ 0.0001 versus vehicle control

So far, C81 has been shown to have promising anti-inflammatory effects on leukocytes. However, successful resolution of inflammation also requires the restoration of functional tissue homeostasis. This involves apoptosis of neutrophils and their subsequent efferocytosis by macrophages [11, 12]. To examine a potential effect of C81 on the apoptosis of neutrophils, PMNLs were isolated from human blood and treated with the indicated concentrations of C81 for 6 h. Fortunately, treatment with already 3 µM of C81 induced apoptosis in neutrophils, as determined by flow cytometry. This effect was further increased at a concentration of 10 µM and was even comparable to the apoptosis level of staurosporine-treated neutrophils (Fig. 6a). In contrast, no induction of apoptosis was observed in M1 and M2 macrophages (Fig. 6b, c), indicating a cell type specific regulation altered by C81. To further distinguish between early and late apoptosis and to exclude C81-induced necrosis of neutrophils, we additionally used Annexin V/PI staining (Fig. 6d). Indeed, the data confirmed the previous conclusion that C81 induced apoptosis in human neutrophils and was determined as early apoptosis. Moreover, as the Annexin V/PI staining assay is a more sensitive method, we already observed a significant increase in apoptosis in neutrophils treated with 0.3 µM C81. More importantly, C81 did not induce necrosis in neutrophils. Notably, we demonstrated that the administration of C81 increased levels of cleaved caspase 3 (i.e. caspase 3 activity) in a concentration dependent manner in neutrophils (Fig. 6e). We then investigated whether the pro-apoptotic effect also results in an increased efferocytosis. For this purpose, monocyte-derived macrophages and C81-treated neutrophils were co-incubated for 1 h, and efferocytosis was quantified by flow cytometry. The results showed that neutrophils pre-treated with C81 are significantly better taken up by macrophages than untreated cells (Fig. 6f).

To summarize, C81 does not only exhibit anti-inflammatory but also enhances the pro-resolving properties, by specifically inducing the apoptosis of neutrophils and by promoting efferocytosis.

### The pharmacological mode of action of C81 relies on the inhibition of the dual-specificity regulated kinase 1B (DYRK1B)

**Fig. 7:**
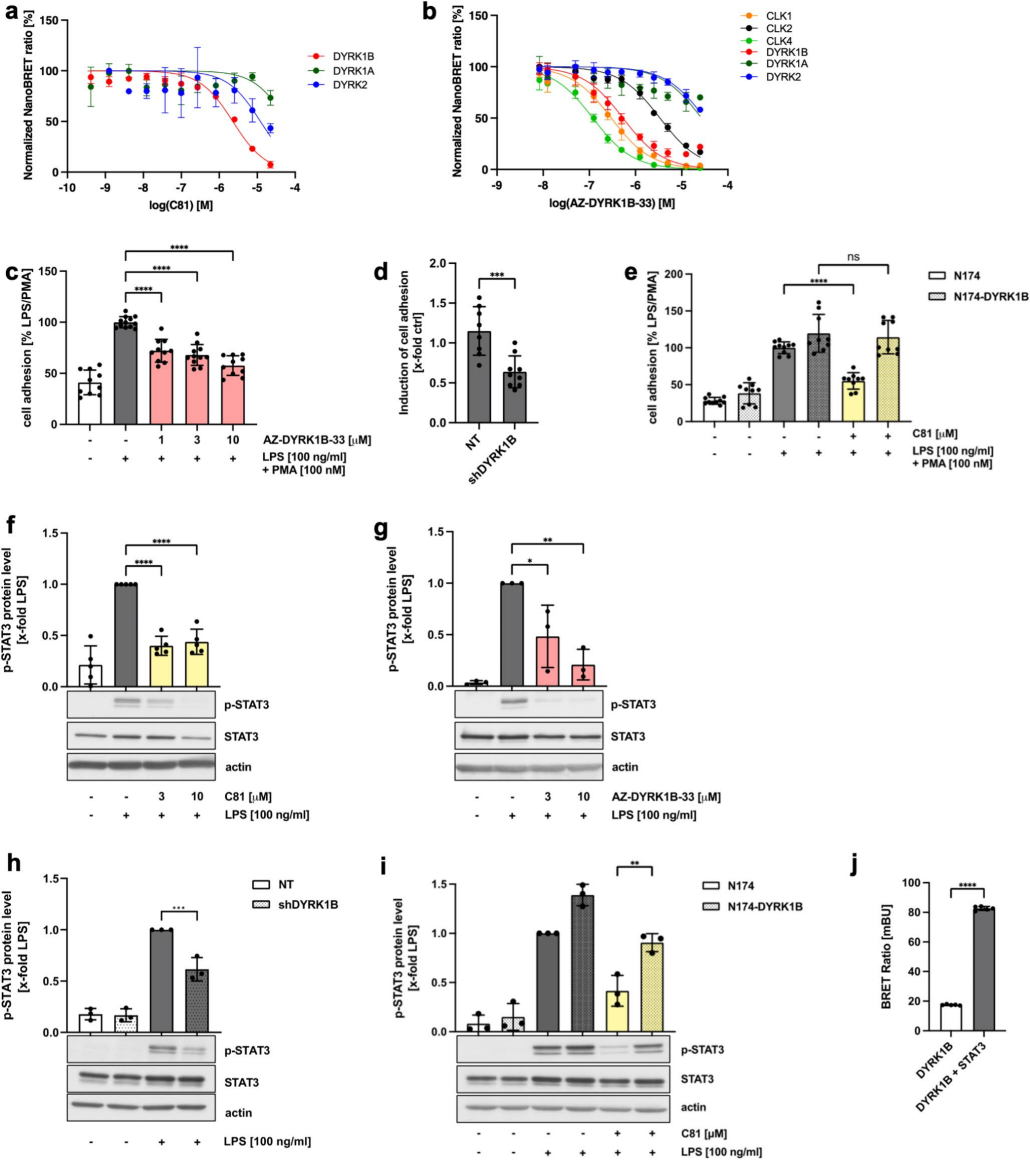
DYRK1B mediates C81-induced effects through phosphorylation of STAT3, as confirmed by DYRK1B knockdown and overexpression. A NanoBRET assay in HEK-293 cells provided apparent EC_50_ values for kinase inhibition by C81 (**a**) and AZ-DYRK1B-33 (**b**). The EC_50_ values were determined for DYRK1B (2.3 µM) and DYRK2 (6.1 µM) for C81 (**a**). For AZ-DYRK1B-33, EC_50_ values were analyzed for DYRK1B (537 nM), DYRK1A (3.8 µM), DYRK2 (3.4 µM), CLK1 (299 nM), CLK2 (5 µM) and CLK4 (119 nM) (**b**). THP-1 cells were pretreated with AZ-DYRK1B-33 or vehicle (DMSO) as indicated for 30 min followed by activation with LPS for 24 h and addition of PMA during the last 10 min of treatment. Cells were fluorescently labeled, added to a confluent HUVEC monolayer and allowed to adhere for 1 h. Adherent cells were quantified by fluorescence measurement (**c**). For knockdown of *DYRK1B*, stably transfected THP-1 cells with a vector containing IPTG-inducible scramble (NT) or shRNA against *DYRK1B* (shDYRK1B) were treated with 1 mM IPTG for 72 h (**d**, **h**). Overexpression of *DYRK1B* was achieved by lentiviral transfection of the vector N174-MCS (Puro) containing the cDNA sequence of *DYRK1B* (N174-DYRK1B). As a control, the N174 vector without cDNA was transfected (N174) (**e**, **i**). Cell adhesion assays were performed as described previously for THP-1 cells (**d**, **e**). The protein level of p-STAT3 over the respective total protein form was determined in M1 macrophages (**f**, **g**) and in stably transfected THP-1 cells (**h**, **i**) by western blot analysis. M1 macrophages were either treated with C81 (**f**), AZ-DYRK1B-33 (**g**) or vehicle (DMSO) for 24 h. One representative blot is shown. Actin was used as loading control (**f-i**). Protein-protein interaction was determined by a NanoBRET assay (**j**). Data are expressed as mean ± SD. (**a**,** b**) *n* = 4, **(c**,** d**,** e**,** h**,** i**) *n* = 3, (**f**,** j**) *n* = 5. Significance calculated by one-way ANOVA followed by Tukey’s post-hoc test (**c**,** e-j**) and unpaired t-test (**d**). *p ≤ 0.05, **p ≤ 0.01, ***p ≤ 0.001, ****p ≤ 0.0001 versus vehicle control

Previously, C81 was identified as a kinase inhibitor: it specifically acts against the CLK family of kinases, which was analyzed in the context of angiogenesis in endothelial cells [17]. To determine the pharmacological mode of action of C81, we aimed at identifying the target kinase(s) involved in the mechanisms of inflammation and its resolution in leukocytes. As Ju et al. had already demonstrated that the inhibition of DYRK1A is able to decrease LPS-mediated neuroinflammation [40] and our group recently has reported that DYRK2 (EC_50_ = 6.1 µM) is a target of C81 [14], we focused here on the interaction of C81 with kinases of the DYRK family in intact cells examined by using a NanoBRET assay. In our experimental setup, we were able to demonstrate that C81 binds most potently to DYRK1B with an EC_50_ of 2.3 µM, while DYRK1A was not bound by C81 (EC_50_ > 50 µM) (Fig. 7a). To confirm an involvement of DYRK1B in the C81-derived effects, we tested an established DYRK1B inhibitor, AZ-DYRK1B-33 [41], for selectivity by again performing a NanoBRET assay in intact cells. AZ-DYRK1B-33 also showed a higher affinity for DYRK1B (EC_50_ = 537 nM) in comparison to DYRK2 (EC_50_ = 3.4 µM) and DYRK1A (EC_50_ = 3.8 µM). Unfortunately, this inhibitor also strongly interacts with kinases of the CLK family, e.g. CLK1 (EC_50_ = 299 nM) and CLK1 (EC_50_ = 119 nM) (Fig. 7b). However, due to the lack of inhibitors with better selectivity and the nevertheless high affinity to DYRK1B, we continueed with AZ-DYRK1B-33 to perform cell adhesion assays as previously described for C81 with THP-1 cells. With this inhibitor, the adhesion of leukocytes on endothelial cells was also strongly inhibited (Fig. 7c).

To further confirm the relevance of DYRK1B, we generated stably transfected THP-1 cells with either an IPTG-inducible knockdown by shRNA or a constitutive overexpression of *DYRK1B*. As a control, we transfected cell lines with a plasmid coding for a non-targeting shRNA or an empty overexpression vector. After confirming downregulation and overexpression of DYRK1B by RT-qPCR (Supplementary Fig. 8), cell adhesion assays were performed. Upon *DYRK1B* knockdown, the LPS/PMA-mediated induction of THP-1 adhesion, which was approximately 1.15fold higher than that of untreated THP-1 cells, was reduced to 0.64-fold (Fig. 7d). In addition, overexpression of DYRK1B largely reversed the inhibitory effect of C81 on cell adhesion, confirming that DYRK1B is a valid target of C81 (Fig. 7e).

To gain deeper insight into the underlying mechanisms, we analyzed potential effects of DYRK1B on STAT3 activation, as DYRK1B has already been shown to directly interact with this transcription factor to induce its phosphorylation [42]. Indeed, binding of DYRK1B by C81 or AZ-DYRK1B-33 strongly inhibited LPS-induced STAT3 phosphorylation in primary human macrophages (Fig. 7f, g). Likewise, an inhibitory effect was observed by shRNA-mediated knockdown of DYRK1B in THP-1 cells (Fig. 7h). Additionally, the C81-evoked reduction in STAT3 activation was mitigated in cell lines overexpressing DYRK1B (Fig. 7i). To verify the direct interaction between DYRK1B and STAT3, we performed a NanoBRET assay, which indicated a protein-protein interaction, as an induced BRET ratio was seen when DYRK1B and STAT3 constructs were co-expressed (Fig. 7j). Additionally, C81 only inhibited LPS-induced STAT3 activation, since the IL-6-mediated phosphorylation was not altered by C81 (Supplementary Fig. 9a). To verify that C81-derived inhibition of STAT3 was indeed independent of IL-6, we blocked IL6R by using tocilizumab and activated the cells with either LPS or IL-6. Whereas IL6R blocking completely abrogated IL-6-mediated STAT3 phosphorylation, LPS still induced STAT3 phosphorylation (Supplementary Fig. 9b) suggesting an IL-6-independent mechanism. Furthermore, after 24 h of LPS treatment an additional activation with IL-6 for 10 min did not result in an increased STAT3 activation (Supplementary Fig. 9c), which indicated IL-6 resistance.

Thus, our data suggested that the pharmacological mode of action of C81 is associated with the inhibition of the protein kinase DYRK1B, which directly mediates an IL-6-independent activation of STAT3.

### STAT3 inhibition by Stattic mimics C81-dependent effects and leads to cell type-specific induction of apoptosis

**Fig. 8:**
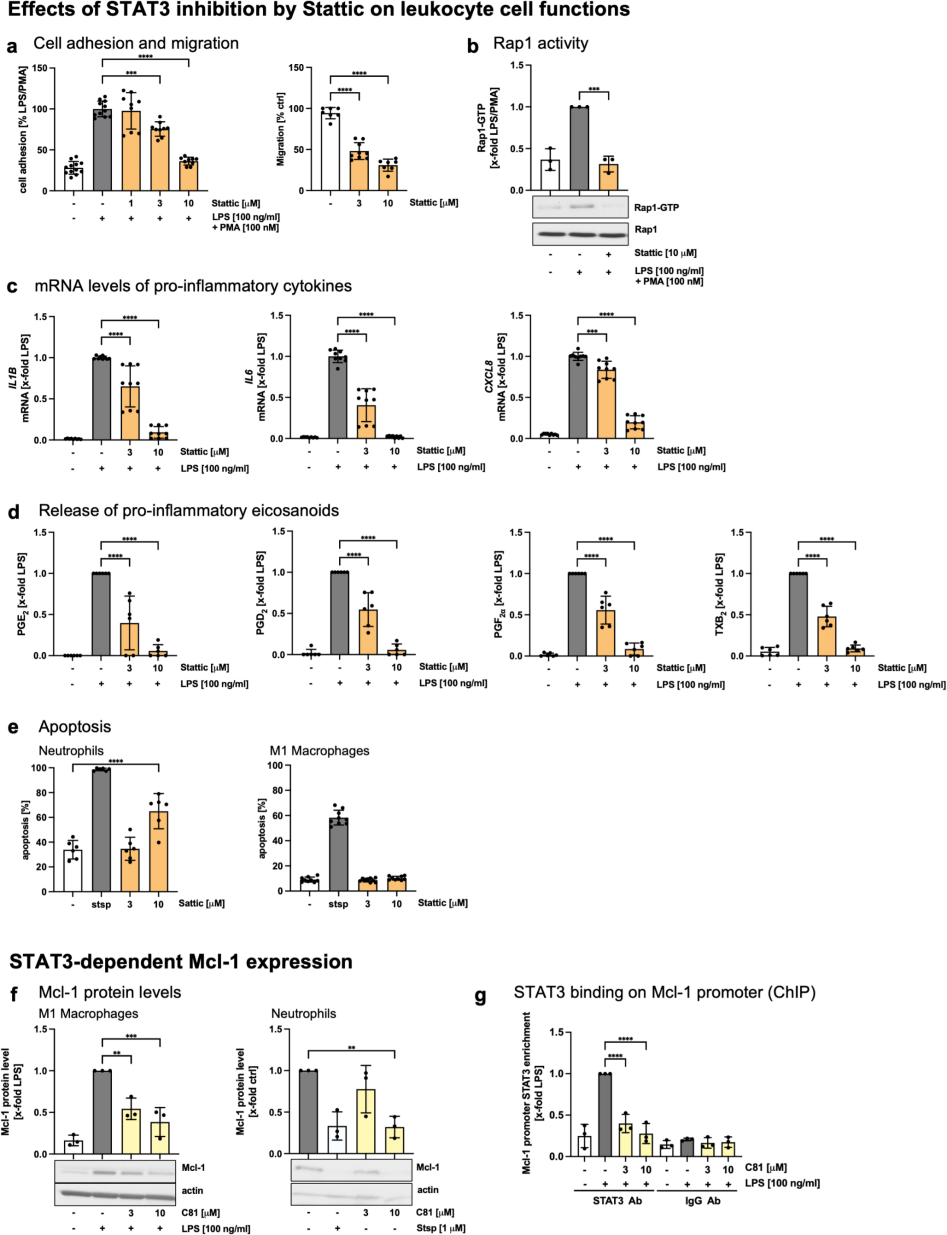
STAT3 inhibition by Stattic mimics C81-derived effects on leukocyte adhesion and migration, cytokine and lipid mediator release and cell-type specific induction of apoptosis by altering Mcl-1 expression. Experiments to determine the effects of Stattic on adhesion of THP-1 cells and undirected migration of macrophages (**a**), Rap1 activity in THP-1 cells (**b**), the mRNA levels of pro-inflammatory cytokines in M1 macrophages (**c**), the release of eicosanoids by M1 macrophages (**d**) and the apoptosis of human neutrophils (**e** left) and macrophages (**e** right) were performed as described previously for C81. Mcl-1 expression was quantified by western blot analysis in M1 macrophages (**f** left) and neutrophils (**f** right). Macrophages were treated with C81 or vehicle (DMSO) as indicated for 30 min followed by LPS for 24 h. Neutrophils were treated with C81 or vehicle (DMSO) for 6 h. Staurosporine (1 µM) was used as a positive control. One representative blot is shown. Actin was used as loading control (**f**). STAT3 binding on Mcl-1 promoter was determined in M1 macrophages by ChIP analysis. For immunoprecipitation, 2 µg of STAT3 antibody and a nonspecific antibody (rabbit IgG) were used. Precipitated DNA was analyzed by qPCR using primers that amplified a region including the STAT3-binding site of human Mcl-1 promoter (**g**). Data are expressed as mean ± SD. (**a-c**,** e-g**) *n* = 3, (**d**) *n* = 6. Significance calculated by one-way ANOVA followed by Tukey’s post-hoc test. **p ≤ 0.01, ***p ≤ 0.001, ****p ≤ 0.0001 versus vehicle control

STAT3 seems to be a key player in C81-mediated effects in leukocytes. To prove the importance of this transcription factor, we made use of the STAT3 inhibitor Stattic [43]. First, we confirmed the potency of the used concentrations of Stattic to reduce STAT3 phosphorylation by Western blotting (Supplementary Fig. 10). Next, we investigated the effect of STAT3 inhibition on cell functions of leukocytes, including adhesion and migration. Interestingly, the adhesion of THP-1 cells as well as the undirected migration of primary human macrophages was strongly reduced in the presence of 3 and 10 µM of Stattic (Fig. 8a). Consistently, treatment with 10 µM Stattic decreased EPAC1 expression (Supplementary Fig. 10b), which completely abolished Rap-1 activity as determined by a Rap1-GTP pulldown assay (Fig. 8b). Additionally, expression of the pro-inflammatory cytokines *IL1B*, *IL6* and *CXCL8* was significantly decreased in the presence of 3 µM Stattic and was diminished upon treatment at a concentration of 10 µM (Fig. 8c). The same effect was observed on the secretion of pro-inflammatory eicosanoids, including PGE_2_, PGD_2_, PGF_2a_ and TXB_2_ as examined by LC-MS/MS (Fig. 8d). As seen before for C81, STAT3 inhibition leads to a reduction of mPGES-1 expression, but more importantly, to a reduction of cPLA_2_ expression. This indicates reduced availability of the prostanoid precursor arachidonic acid (Supplementary Fig. 10c, d). Surprisingly, Stattic strongly induced apoptosis of human neutrophils without affecting cell viability of macrophages (Fig. 8e). To explain this cell type-specific induction of apoptosis, we analyzed the STAT-mediated expression of Mcl-1 by Western blotting. In fact, Mcl-1 expression was significantly reduced in macrophages and neutrophils after C81 treatment (Fig. 8f). By ChIP analysis, we demonstrated diminished binding of STAT3 to the *MCL1* promoter in the presence of 3 and 10 µM C81 (Fig. 8g). MCL-1 is known to be an important anti-apoptotic mediator, but it has been shown to be essential for the survival of neutrophils rather than macrophages [44].

In summary, modulation of STAT3 signaling by Stattic mimicked C81-derived anti-inflammatory and pro-resolving effects on leukocytes, including leukocyte diapedesis, pro-inflammatory cytokine and lipid mediator release and cell type-specific regulation of apoptosis.

## Discussion

Inflammation is mediated by the activation of inflammatory signaling pathways, such as NFκB, MAPK and JAK-STAT. These pathways are initiated by ligand-receptor binding and subsequent signal transduction and activation of transcription factors regulating the expression of genes involved in the inflammatory response. Thus, the transcription factor STAT3 is directly associated with the IL-6/JAK-STAT axis [45]. In this study, we focused on the impact of the alkaloid derivative C81 on the inflammatory response and its resolution in vivo and in vitro and demonstrated the importance of STAT3 in DYRK1B-mediated leukocyte inflammatory processes, including migration, adhesion, release of pro-inflammatory mediators and cell-type specific regulation of apoptosis.

We demonstrated strong anti-inflammatory and pro-resolving effects of C81 in vivo in an imiquimod-induced psoriasis mouse model. The development of disease-related symptoms, such as redness, scaling and thickness, was strongly inhibited and resolved more quickly upon C81 treatment compared to the control. Additionally, we found that C81 reduces the infiltration of microglia and macrophages in the laser lesion area in a CNV model in vivo, suggesting reduced cell migration capability and/or impaired leukocyte-endothelial cell interaction. Indeed, in vitro analysis demonstrated that C81 inhibited the undirected as well as directed serum-induced migration of primary human macrophages. Furthermore, we could also show that C81-treated human monocytes were prevented from adhering to and transmigrating through endothelial cells. This was in line with previously published data, demonstrating that C81 reduced leukocyte extravasation in vivo, in particular adhesion and transmigration, as analyzed by intravital microscopy in a murine cremaster model. Previous studies focused on the anti-inflammatory impact of C81 in endothelial cells and demonstrated compound-derived inhibition of TNF-induced cell adhesion molecule (CAM) expression [14]. In leukocytes, migration and adhesion relies on the conformational change of constitutively expressed integrins, which is mediated by the small GTPase Rap1 [46]. Previously, it was shown that Rap1 and consequently β1 integrin activation is induced by specifically activating cAMP-dependent EPAC1 in U937, a pro-monocytic cell line, and primary human monocytes. Thereby, cell adhesion and chemotaxis were significantly upregulated proving the importance of EPAC1-Rap1 signaling in monocytes [39]. Our data support these findings, as C81 abolished Rap1-dependent integrin activation in THP-1 cells due to reduced *RAPGEF3* mRNA and consequently dampened EPAC1 protein expression. Additionally, we demonstrated that STAT3 inhibition by Stattic mimicked the inhibitory effect of C81 on monocyte adhesion and migration by reducing EPAC1 expression, leading to complete inhibition of Rap1 activation. We therefore suggest that STAT3 is involved in the regulation of EPAC1 expression, about which little is known. Though, EPAC1 is assumed to negatively regulate STAT3 activation [47–49]. In regulatory T cells (T_reg_), EPAC1 knockdown or inhibition has been shown to result in increased STAT3 phosphorylation and decreased T_reg_-mediated suppression of effector T cell (T_eff_) proliferation. Furthermore, STAT3 inhibition also suppresses T_eff_ proliferation, leading to the assumption that EPAC1 activity or expression is induced, which was not further investigated [49]. Nevertheless, our data clearly demonstrated that EPAC1 expression is STAT3-dependent.

Importantly, we found that C81 treatment resulted in a strong inhibition of the LPS-induced mRNA expression of pro-inflammatory cytokines, including *IL6*, *IL1B* and *CXCL8*, in monocyte-derived M1 macrophages. As we were able to phenocopy this effect by suppressing STAT3 activation, we assumed that the expression of these cytokines was mediated by the STAT3 pathway. Indeed, Samavati et al. have shown that expression of IL-1β and IL-6, induced by LPS, was significantly reduced upon STAT3 inhibition in the murine macrophage cell line RAW 264.7 [50]. In addition, suppressed STAT3 activation inhibited LPS-induced IL-8 expression in THP-1 cells differentiated into macrophages [51]. In contrast, *CXCL8* mRNA expression was not reduced in C81-treated M2 macrophages. As IL-8 promotes cancer progression, it has been extensively investigated in this context and revealing interference with STAT3 signaling [52–56]. Tumor-associated macrophages (TAMs), which predominantly exhibit the M2 phenotype, are known to mainly release IL-8, which promotes the recruitment of cancer cells to the tumor microenvironment [57–60]. Thus, our results not only identified the dependence of pro-inflammatory cytokine expression on STAT3 activation, but also indicated that the regulation of IL-8 expression in M2 macrophages is different from that in M1 macrophages.

Surprisingly, our data revealed that C81 inhibited the LPS-induced secretion of pro-inflammatory eicosanoids, including PGE_2_, PGD_2_, TXB_2_ and PGF_2a_ in macrophages. Of note, PGD_2_ levels remained consistently low in M2 macrophages even after LPS stimulation, which to our knowledge has not been reported previously. The biosynthesis of eicosanoids is rate limited by the activity of cPLA_2_, which hydrolyzes membrane phospholipids to release arachidonic acid [61]. In 2006, a cPLA_2_ inhibitor (NCT00396955) reached a phase II trial for the treatment of rheumatoid arthritis, but unfortunately the study was terminated due to adverse effects. However, extensive research continued for the development of inhibitors, which have been demonstrated to promisingly reduce the inflammatory response [62–67]. In addition, cPLA_2_ was also found to be involved in adipogenesis, and, interestingly, a double-negative mutant of STAT3 inhibited cPLA_2_ expression in preadipocytes [68, 69]. This supports our findings, as STAT3 inhibition in macrophages reduced cPLA_2_ expression and abolished eicosanoid secretion. Data of Sommerfelt et al. indicated a reduction of COX-2 expression in TLR2 signaling when cPLA_2_ was inhibited [64]. In contrast, we have found an induced COX-2 expression by C81-mediated cPLA_2_ inhibition, suggesting some sort of compensatory effects. Research into NSAIDs, the main drugs used for pain relief, has shown that COX inhibition can have serious side effects, including gastrointestinal and cardiovascular problems [70, 71], so the focus is now shifting to alternatives such as targeting mPGES-1 [72–75]. Inhibition of mPGES-1 has been associated with enhanced release of pro-resolving PGD_2_ metabolites and a reduced risk of cardiovascular side effects [74, 75]. In our study, we observed a dramatic inhibition of mPGES-1 expression upon C81 treatment due to STAT3 inhibition. A link between mPGES-1 and STAT3 has been suggested in the literature, e.g. inhibition of mPGES-1 reduces STAT3 phosphorylation [76], mPGES-1-derived PGE_2_ promotes STAT3 activation [77], and IL-6 induces mPGES-1 expression, which is associated with STAT3 activity in the rat brain [78]. However, our data indicated STAT3-dependent mPGES-1 expression, which to our knowledge has not been investigated and should be considered in the future.

Neutrophils are the most abundant immune cells in human blood and are the first to enter inflamed tissue to contribute to host defense with their potent antimicrobial activity [79–82]. As a result, they are primarily responsible for tissue damage once the inflammatory stimuli have been eliminated, and therefore their lifespan needs to be tightly regulated [83]. Resolution of inflammation was significantly upregulated when neutrophil apoptosis was promoted by treatment with IFNβ, a CDK inhibitor and 15-epi-lipoxin A_4_ [84–86]. Here we report a C81-derived cell-type specific upregulation of apoptosis in human neutrophils, without inducing necrosis and without affecting cell viability of other primary human leukocytes, including monocytes and M1 and M2 macrophages. According to the studies on IFNβ, CDK inhibitor and 15-epi-lipoxin A_4_-induced neutrophil apoptosis, the mode of action of C81 was based on the reduced expression of the anti-apoptotic mediator Mcl-1, resulting in the activation of caspase 3, which leads to apoptotic cell death [85–87]. Unlike neutrophils, the survival of monocytes and macrophages is not dependent on Mcl-1, allowing selective induction of apoptosis [44]. Our data showed that *MCL1* gene expression was highly dependent on STAT3, as Mcl-1 expression on protein level and LPS-induced STAT3 binding to the *MCL1* promoter were abolished by C81-derived STAT3 inhibition. Our findings are supported by data from Isomoto et al., who previously demonstrated direct STAT3 binding to the *MCL1* promoter by chromatin immunoprecipitation in a cancer cell line [88]. Furthermore, in human neutrophils inhibition of the JAK-STAT pathway decreased Mcl-1 expression, and IFNβ-induced Mcl-1 depletion was also associated with STAT3 activity [86, 89]. In LPS-mediated lung inflammation, the pharmacological inhibition of Mcl-1 did not only result in induced apoptosis but also induced efferocytosis [90]. This is consistent with our data, as C81-induced neutrophil apoptosis significantly promoted the uptake by macrophages. This complements C81’s strong anti-inflammatory effects of C81 with promising pro-resolving properties already demonstrated in vivo.

However, imiquimod induces psoriasis largely through activation of TLR7 [24]. While TLR4 can be activated from the cell surface and the endosome and induces myeloid differentiation primary response 88 (MyD88)- and toll-interleukin 1 receptor (TIR) domain-containing adapter-inducing interferon-β (TRIF)-dependent pathways, TLR7 is located predominantly in endosomes [91]. Here, TLR7 is usually triggered by ssRNA derived from viruses, resulting in the activation of MyD88 [91]. Crucially, while MyD88 activation from TLR4 requires TIR domain containing adaptor protein (TIRAP) [92], TLR7 was initially thought to be TIRAP independent [93]. However, a more recent study found that full activation of TLR7-dependent MyD88 signaling also requires TIRAP recruitment [94]. Moreover, some data suggests that TLR7 might also be capable of activating TRIF [95]. Therefore, while there are differences in TLR4 and TLR7 induced signaling, they are mostly minor. While this could still indicate that C81 derived effects might be obtained from distinct mechanisms in the respective in vivo model, we believe it is reasonable to assume that the roles of C81 and DYRK1B in TLR4 and TLR7 signaling are likely similar. Nonetheless, C81, other DYRK1B inhibitors such as AZ-DYRK1B-33, and DYRK1B knockdowns should be applied in TLR7 dependent models, to fully elucidate their mechanism of action in inflammation derived from TLR7 activation.

Finally, the involvement in the inflammatory response in leukocytes of the kinase DYRK1B is worth highlighting. We identified DYRK1B as a target of C81 by BRET based target engagement assays and we demonstrated its relevance in inflammatory processes. Thereby, we found that C81-derived reduction of cell adhesion was mediated by DYRK1B, as pharmacological inhibition with the potent DYRK1B inhibitor AZ-DYRK1B-33 and knockdown phenocopied the effect and overexpression attenuated the inhibitory effect. Recently, inhibition of DYRK1B was reported to reduce inflammation in an allergic contact dermatitis model by using AZ-DYRK1B-33 [96]. This supports our finding that C81-mediated DYRK1B inhibition in an imiquimod-induced psoriasis model results in reduced inflammation and induced resolution. Furthermore, our data demonstrate DYRK1B to be essential for LPS-induced activation of STAT3, as pharmacological DYRK1B inhibition and knockdown reduced STAT3 phosphorylation, which was mitigated upon DYRK1B overexpression. Additionally, we found that STAT3 activation upon LPS treatment is IL-6-independent. Meley et al. already have shown in dendritic cells that IL-6 is not involved in the phosphorylation of STAT3 upon LPS stimulation. Interestingly, they found that ERK1/2 inhibition leads to induced phosphorylation of STAT3 [97]. Independent of this study, DYRK1B is known to be an ERK2 substrate and its activity is reduced by ERK2 inhibition, but on the other hand, DYRK1B expression is induced by inhibition of MEK1/2-ERK1/2 [98–100]. Higher levels of DYRK1B upon ERK1/2 inhibition may explain the induced STAT3 phosphorylation seen in Meley’s study. As we also found an augmented STAT3 phosphorylation by overexpressing DYRK1B, we suggest further investigations of ERK-DYRK1B interaction to reveal still unknown upstream mechanisms. This is the first time that DYRK1B together with STAT3 are proposed to be important players in the context of inflammation. An interaction between these two proteins has already been described in cardiac hypertrophy and heart failure, where DYRK1B overexpression directly correlated with increased activation of STAT3. We also demonstrated a direct interaction between DYRK1B and STAT3, supporting the findings of Zhuang et al. that DYRK1B directly phosphorylates STAT3 [42]. STAT3 is involved in multiple cellular processes and was also described to have cardioprotective effects and additionally can promote resolution of inflammation when activated by IL-10 [101–104]. Dysregulated, including increased and decreased, activation or expression of STAT3 is associated with various human diseases, requiring a fine-tuning of STAT3 modulation [105]. Our data demonstrated that targeting DYRK1B to reduce STAT3 activity represents a new and promising future perspective for the treatment of chronic inflammatory diseases. However, it should be considered that developing selective DYRK1B inhibitors is very challenging due to the high similarity of kinases belonging to the CMGC family, including CLKs as well as the other DYRKs [18, 20, 106]. As we have shown, the proposed selective inhibitor AZ-DYRK1B-33 [41] and our compound C81 also interacted strongly with CLK1, CLK2, CLK3 and CLK4, but also with DYRK2, indicating a high risk of unwanted side effects. Notably, the pan-CLK DYRK inhibitors lorecivivint (NCT04520607) and cirtuvivint (NCT05084859) have entered clinical trials for the treatment of knee osteoarthritis and cancer, respectively. Looking to the future, the development of more selective inhibitors could open up the possibility of treating numerous diseases.

Taken together, using the alkaloid-derived compound C81, we uncovered DYRK1B/STAT3 axis as a new regulator of inflammatory processes in human leukocytes. Interfering with the DYRK1B-STAT3 signaling can address essential cell functions including leukocyte extravasation, pro-inflammatory mediator release, neutrophil apoptosis and efferocytosis. Furthermore, using two different mouse models, we were able to demonstrate the in vivo relevance of this signaling axis and highlight DYRK1B as an important kinase regulating inflammation and resolution.

## Electronic supplementary material

Below is the link to the electronic supplementary material.

Supplementary Material 1

## Data Availability

All data supporting the findings are available in the main text and the supplementary file. Other data that supports the findings of this manuscript can be obtained from the corresponding author upon request.
