# Supplementary material for: Inhibition of DYRK1B BY C81 impedes inflammatory processes in leukocytes by reducing STAT3 activity
Source: Cell Mol Life Sci. 2025 Feb 22;82(1):85. doi: 10.1007/s00018-025-05579-y (PMC11846820; doi:10.1007/s00018-025-05579-y)
Supplement: Supplementary file 1 — Supplementary Material 1 [file 18_2025_5579_MOESM1_ESM.docx]

DYRK1B/STAT3 SIGNALING AS A NEW REGULATOR OF INFLAMMATORY PROCESSES IN LEUKOCYTES

Ciurus et al.

**Supplementary Figures**

**
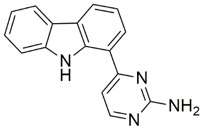
**

**Supplementary Figure 1** Chemical structure of C81.

**Supplementary Figure 2** C81 has no effect on the weight change curve in an imiquimod-induced psoriasis mouse model. Daily application of imiquimod treatment started on day 0 for 6 consecutive days. C81 (1 mg/kg) or vehicle (10 % DMSO, 90 % PBS/Kolliphor EL (90 %/10 % [v/v]) were injected subcutaneously on days 1, 3 and 5. Mice were weighed daily. Data are expressed as mean ± SD. Significance calculated by two-way ANOVA followed by Sidak’s multiple comparison test.

**Supplementary Figure 3** Even a lower dose of C81 (3 μM) significantly decreases infiltration of Iba1^+^ mononucelar phagocytes in the RPE in laser-induced choroidal neovascularization (CNV) model *in vivo*. C81 (3 μM, end concentration in the eye) or vehicle (0.05 % DMSO) was injected intravitreally immediately after laser pulse application. After 7 days, morphological changes (**b**) and infiltration (**c**) of Iba1^+^ cells were quantified in retinal and RPE/choroidal flat mounts by immunohistochemistry and fluorescence microscopy (**a**). Scale bar: 50 μm. One representative experiment is shown. n = 34-46 retinal spots and n= 30-44 RPE/choroidal spots. Data are expressed as mean ± SD. Significance calculated by unpaired t-test. **p ≤ 0.001 versus vehicle control for three individual animals.

**Supplementary Figure 4** C81 does not impair cell viability of leukocytes up to 10 μM. The effect of C81 on the metabolic activity (**a**), membrane integrity (**b**), apoptosis (**c**) and cell viability (**d**) was determined. THP-1 cells and primary human leukocytes, including monocytes and monocyte-derived M1 and M2 macrophages were stimulated for 24 h with the indicated concentrations or DMSO (0.01%) and the respective cytotoxicity assay was performed. Data are expressed as mean ± SD. n=3. Significance calculated by one-way ANOVA followed by Tukey’s post-hoc test.

**Supplementary Figure 5** C81 inhibits undirected migration and activation of STAT3 in M2 macrophages. For undirected migration, a confluent layer of monocyte-derived M2 macrophages was treated with C81 or vehicle (DMSO) and a scratch assay was performed (**a**). M2 macrophages were activated with 100 ng/ml LPS after 30 min pre-treatment with C81 or vehicle (DMSO). The protein level of phospho-STAT3 and of total STAT3 were determined by western blot analysis. Actin was used as loading control (**b**). One representative blot is shown. Data are expressed as mean ± SD. n=3. Significance calculated by one-way ANOVA followed by Tukey’s post-hoc test. ****p ≤ 0.0001 versus vehicle control.

**Supplementary Figure 6** C81 has only a minor effect on the cell surface expression of VLA-4. Primary monocytes were stimulated with the indicated concentrations of C81 or DMSO for 24 h. The cell surface expression of LFA-1 (**a**), Mac-1 (**b**) and VLA-4 (**c**) was analyzed by flow cytometry. Data are expressed as mean ± SD. n=3. Significance calculated by one-way ANOVA followed by Tukey’s post-hoc test. *p ≤ 0.05, ****p ≤ 0.0001 versus vehicle control.

**Supplementary Figure 7** C81 reduces *RAPGEF3* expression on mRNA level. THP-1 cells were activated with LPS for 24 h after a 30 min pre-treatment with C81 or vehicle (DMSO). mRNA expression of *RAPGEF3* was determined by RT-qPCR. GAPDH was used as housekeeping gene. Data are expressed as mean ± SD. n=3. Significance calculated by one-way ANOVA followed by Tukey’s post-hoc test. ****p ≤ 0.0001 versus vehicle control.

**Supplementary Figure 8** *DYRK1B* expression is downregulated in IPTG-treated stably transfected THP-1 cells expressing shDYRK1B and upregulated in stably transfected THP-1 cells expressing the cDNA sequence of *DYRK1B.* For *DYRK1B* knockdown and overexpression, stably transfected THP-1 cells were generated with IPTG-inducible vectors containing non-targeting shRNA (NT) or against human *DYRK1B* (**a**) and lentiviral N174-MCS (puro) vector with (N174-DYRK1B) or without (N174) *DYRK1B* cDNA (**b**). Downregulation of DYRK1B expression was achieved by treatment with 1 mM IPTG for 72 h (**a**). mRNA expression of *DYRK1B* was determined by RT-qPCR. GAPDH was used as housekeeping gene. Data are expressed as mean ± SD. n=3. Significance calculated by one-way ANOVA followed by Tukey’s post-hoc test. ****p ≤ 0.0001 versus vehicle control.

**Supplementary Figure 9** LPS-induced STAT3 phosphorylation is IL-6 independent. M1 macrophages were activated with 50 ng/ml of LPS after 30 min pre-treatment with C81 or vehicle (DMSO) (**a**). M1 macrophages were pre-treated with 25 μg/ml of tocilizumab for 30 min and 100 ng/ml of LPS or 50 ng/ml of IL-6 was added for 2.5 h (**b**). M1 macrophages were activated with 100 ng/ml of LPS for 24 h. 50 ng/ml of IL-6 was added for 10 min (**c**). The protein level of p-STAT3 over the respective total protein form was determined by western blot analysis. Actin was used as loading control (**a-c**). One representative blot is shown. Data are expressed as mean ± SD. n=3. Significance calculated by one-way ANOVA followed by Tukey’s post-hoc test. ****p ≤ 0.0001 versus vehicle control.

**Supplementary Figure 10** Stattic inhibits STAT3 phosphorylation and decreases *RAPGEF3*, *PLA2G4A* and mPGES-1 expression. M1 macrophages were activated with 100 ng/ml of LPS after 30 min pre-treatment with Stattic or vehicle (DMSO) (**a-d**). The protein levels of phospho- and total STAT3 were determined by western blot analysis (**a**). Expression of *RAPGEF3* (**b**) and *PLA2G4A* (**c**) on mRNA and protein level were determined by RT-qPCR and western blot analysis, respectively. The protein level of phospho- and total cPLA_2_ were quantified (**c**). mPGES-1 expression was analyzed by western blot analysis (**d**). B2M served as housekeeping gene. Actin was used as loading control (**a-d**). One representative blot is shown. Data are expressed as mean ± SD. n=3. Significance calculated by one-way ANOVA followed by Tukey’s post-hoc test. *p ≤ 0.05, **p ≤ 0.01, ***p ≤ 0.001, ****p ≤ 0.0001 versus vehicle control.
